# Antigenic and Substrate Preference Differences between Scorpion and Spider Dermonecrotic Toxins, a Comparative Investigation

**DOI:** 10.3390/toxins12100631

**Published:** 2020-10-01

**Authors:** Ramla Ben Yekhlef, Liza Felicori, Lucianna Helene Santos, Camila F. B. Oliveira, Raoudha Fadhloun, Elham Torabi, Delavar Shahbazzadeh, Kamran Pooshang Bagheri, Rafaela Salgado Ferreira, Lamia Borchani

**Affiliations:** 1 Laboratoire des Venins et Biomolécules Thérapeutiques LR16IPT08, Université de Tunis El Manar, Institut Pasteur de Tunis, Tunis 1002, Tunisia; by.ramla@yahoo.fr (R.B.Y.); fadhloun.raoudha@gmail.com (R.F.); 2 Departamento de Bioquímica e Imunologia, Universida de Federal de Minas Gerais, Belo Horizonte, Minas Gerais 31270-901, Brazil; liza@icb.ufmg.br (L.F.); luciannahss@gmail.com (L.H.S.); camilafrancobo@gmail.com (C.F.B.O.); rafaelasalgadoferreira@gmail.com (R.S.F.); 3 Venom and Biotherapeutic Molecules Lab., Medical Biotechnology Department, Biotechnology Research Center, Pasteur Institute of Iran, Tehran 13169-43551, Iran; e.torabi88@gmail.com (E.T.); shahbazzadeh@yahoo.com (D.S.); kamranpb@gmail.com (K.P.B.)

**Keywords:** *Hemiscorpius*, *Loxosceles*, phospholipase D, transphosphatidylase activity, cyclic phosphatidic acid

## Abstract

The *Hemiscorpius lepturus* scorpion and brown spider *Loxosceles intermedia* represent a public health problem in Asia and America, respectively. Although distinct, these organisms contain similar toxins responsible for the principal clinical signs of envenomation. To better understand the properties of these toxins, we designed a study to compare recombinant Heminecrolysin (rHNC) and rLiD1, the major phospholipase D toxins of scorpion and spider venom, respectively. Using a competitive ELISA and a hemolytic inhibition test, we come to spot a cross reaction between scorpion and spider venoms along with an epitopic similarity between rHNC and rLiD1 associated with neutralizing antibodies. Results show that the ability of the rHNC to hydrolyze lysophosphatidylcholine (LPC) is equivalent to that of rLiD1 to hydrolyze sphingomyelin and vice-versa. rHNC exclusively catalyze transphosphatidylation of LPC producing cyclic phosphatidic acid (cPA). The in-silico analysis of hydrogen bonds between LPC and toxins provides a possible explanation for the higher transphosphatidylase activity of rHNC. Interestingly, for the first time, we reveal that lysophosphatidic acid (LPA) can be a substrate for both enzymes using cellular and enzymatic assays. The finding of the usage of LPA as a substrate as well as the formation of cPA as an end product could shed more light on the molecular basis of *Hemiscorpius lepturus* envenomation as well as on loxoscelism.

## 1. Introduction

*Hemiscorpius lepturus* is considered as the most medically important and dangerous scorpion in Khuzestan, Iran, and the world. Thus, it has been responsible for 15% of the scorpion sting bite cases, leading to a death rate reaching 89%. The lethality arising from this scorpion is approximately 60 times higher than the average for the remaining venomous scorpion stings in the region [1]. Loxosceles spiders are arachnids that are widely distributed around the globe in tropical and temperate regions. Several reports notified Brazil and America, particularly its south part, as the most concerned regions by loxoscelism. In 2019, 8490 *Loxosceles* bites were recorded only in Brazil [2]. *Hemiscorpius lepturus* (*H. lepturus*) scorpion and *Loxosceles* (*L*.) brown spiders share several clinical and toxicological-induced manifestations such as hemolysis, local skin injury, vascular leakage, persistent inflammation, platelet aggregation, and acute renal failure [1,3,4]. Although these venoms contain several proteins, sphingomyelinases D (SMaseD) are responsible for their major physiopathological effects [5,6].

Spider’s SMaseD proteins are very characteristic and conserved molecules. They owe their name to their ability to hydrolyze sphingomyelin (SM) [7]. Later they were found to hydrolyze not only SM but also lysophosphatidylcholine (LPC), producing ceramide-1-phosphate and lysophosphatidic acid (LPA), respectively, as a second product. They were therefore renamed Phospholipases D (PLD) to represent a broader specificity [8,9]. Lastly, by using ^31^P NMR and mass spectrometry, Lajoie et al., 2013 [10], showed that PLD spiders catalyze preferentially a transphosphatidylation reaction, forming cyclic ceramide (1,3) phosphate and cyclic phosphatic acid (cPA) from SM and LPC substrates, respectively.

Several studies have reported that SMases D differ in their catalytic efficiency, substrate specificity, or the intensity of their biological effects. These molecules have been classified into two classes based on sequence, structural, and biochemical data [11]. Class I, represented by SMaseD1 from *Loxosceles laeta*, is characterized by the presence of a single disulfide bridge and an extended hydrophobic loop. Class II comprises SMasesD that contain an additional intra-chain disulfide bridge linking the flexible loop to the catalytic loop. Depending on their ability to hydrolyze SM, they were further subdivided into class IIa, more active, and IIb, less active or inactive [12]. Several spiders’ SMasesD isoforms have already been described with similarity levels varying from 99% to 55% [13]. Studies have shown that the recombinant form of *L. intermedia* dermonecrotic protein (rLiD1), one of the most abundant SMaseD in *L. intermedia* venom, presents dermonecrotic and hemorrhagic activities in rabbits [14,15]. It was also observed that antibodies raised against this class II SMaseD toxin or its epitopes were able to neutralize either rLiD1 or the whole venom in vivo [16,17,18].

On the other hand, Heminecrolysin (HNC) is the only scorpion’s SMaseD purified from the venom of *H. lepturus* [6]. It was considered, structurally and pathophysiologically, as a venom PLD (vPLD). Its physiopathological effects have been shown to correlate with its lysoPLD activity [19]. Furthermore, HNC was found to be highly immunogenic and able to raise antibodies that efficiently neutralize whole venom effects in vitro and in vivo [20].

The *H. lepturus* transcriptomic study revealed that, similarly to spiders, different isoforms of PLD are detected in the venom [21]. The isoform KY287766 (Hl-PLD1) was cloned, expressed, and purified as the first recombinant *H. lepturus* dermonecrotic toxin. Hl-PLD1 has been reported to have the highest sequence similarity with the PLD isoforms from *L. intermedia* (identity 46–48%) [22].

We designed this study to compare the antigenic and the biochemical properties of the unique scorpion PLD, Hl-PLD1, considered in this study as the recombinant form of HNC (rHNC), and the *L. Intermedia* spider’s PLD, rLiD1. We investigated their immuno cross-reactivity, their substrates preference and their transphosphatidylation activity. The difference in substrate preference of these enzymes was exploited by molecular dynamics and validated in vitro using two breast cancer cell lines expressing different sensitivity to LPC: MDA-MB-231, a highly aggressive and metastatic breast cancer cells, whose proliferation is not affected by LPC and MCF7, a barely invasive and metastatic breast cancer cell, sensitive to LPC.

## 2. Results

### 2.1. Cross-Reactivity of Anti-H. Lepturus and Anti-Loxosceles against rHNC and rLiD1

The cross reactivity of anti-*Loxosceles* and anti-*H. lepturus* (anti-HL) anti-sera as well as anti-rLiD1 (anti-serum against the major dermonecrotic toxin from *L. intermedia* venom) were evaluated against *H. lepturus* venom and the scorpion and spider recombinant toxins (rHNC, rLiD1) by ELISA.

**Figure 1:**
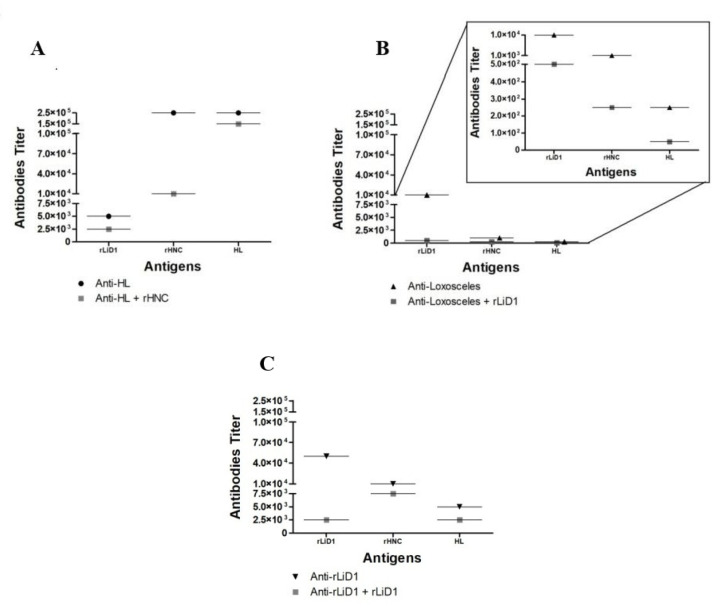
Comparison of the antibody titer of anti-scorpion and spider anti-venoms and anti-toxins against spider (rLiD1) and scorpion toxin (rHNC) and *Hemiscorpius lepturus* (HL) venom. Reactivity against anti-HL (**A**), anti-*Loxosceles* (**B**), anti-rLiD1 (**C**) antibodies. The antibody titer was considered as the highest dilution of serum capable of resulting in a reading greater than 1.0. For this calculation, eight different dilutions of each one of the antibodies (anti-HL, anti-*Loxosceles* and anti-rLiD1) were used (1:100, 1:500, 1:1000, 1:5000, 1:10,000, 1:50,000, 1:100,000, 1:250,000). For competition ELISA, the different antigens (10^−6^ M) were incubated for 30 min at 37 °C with each dilution of each antibody.

Besides the whole venom and the scorpion venom toxin, we perceived that anti-HL recognizes rLiD1 toxin as well (Figure 1A). Competitive ELISA showed that scorpion toxin rHNC shared similar epitopes with rLiD1, since pre-incubation of anti-HL with rHNC decreased the reactivity of anti-HL with rLiD1. Anti-*Loxosceles* anti-venom was also reactive against HL and rHNC (Figure 1B). It was also observed that pre-incubation of anti-*Loxosceles* anti-venom with rLiD1 decreased the reactivity of this anti-venom against HL and rHNC, showing one more time that rLiD1 shares similar epitopes with *H. lepturus* venom in addition to its major toxin. Finally, it was also observed that anti-rLiD1 antibodies recognize likewise rLiD1, HL, and rHNC (Figure 1C).

### 2.2. Cross-Neutralization of Complement-Dependent Hemolytic Activity

**Figure 2:**
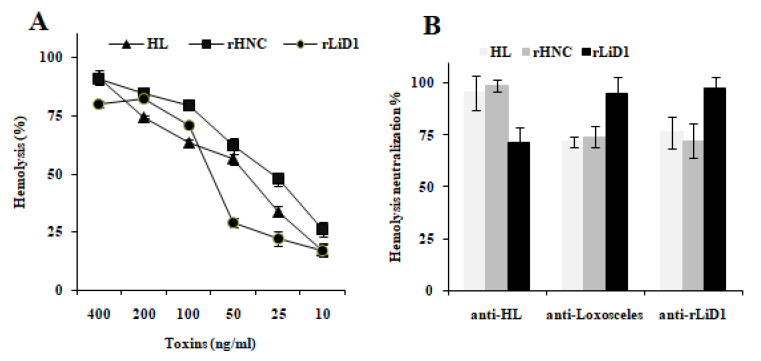
Neutralization of HL venom- rHNC- and rLiD1-induced hemolytic effect by immune sera. (**A**) Whole *H. lepturus* venom (HL), rHNC and rLiD1 were tested from 400 to 10 ng/mL for their capacity to lyse human RBCs in the presence of control-matched serum as a source of complement. Results are expressed as percentages of hemolysis. (**B**) Neutralization of RBCs hemolysis by anti-HL, anti-*Loxosceles,* and anti-rLiD1 immune sera. Samples were mixed with diluted anti-sera (1:50) and incubated with 2% RBCs. Percentages of neutralization were determined as described in methods. Results are representative of three different experiments expressed as the mean of duplicates ± tandard deviation.

Venoms from the spiders *Loxosceles* and *H. lepturus* are known by their ability to sensitize human RBCs to lysis by the autologous complement. We choose this characteristic to determine the cross-neutralization activity of both venoms. Hence, we determined if inhibition of *H. lepturus*, rHNC or rLiD1 by the immune sera (anti-HL, anti-Loxosceles and anti-rLiD1) could neutralize venom- or toxins-induced hemolysis. The results previously published by Torabi et al., 2017 [22] described rHNC only as a dermonecrotic toxin not endowed with hemolytic activity. This led us to first verify its hemolytic potential. We also checked that *H. lepturus* venom and rLiD1 preparation utilized in this study induced RBCs hemolysis. Figure 2A shows that rHNC sensitizes erythrocytes to lysis by autologous complement in a dose dependent manner. The concentration of 25 ng/mL causing 50% hemolysis (EC_50_) of 2% human erythrocytes was correspondingly found with the native toxin [6]. At the same experimental conditions, rLiD1 toxin and *H. lepturus* crude venom displayed a hemolytic activity with an EC_50_ of about 75 ng/mL and 40 ng/mL respectively. The discrepancies between our results and those of Torabi et al., 2017 [22] are most likely due to the absence in their experimental assays conditions of Mg^2+^ and Ca^2+^, two metal cations required for enzyme activity.

Aiming to study the cross neutralization of hemolytic activity of spider *Loxosceles* and *H. lepturus* scorpion venoms by the autologous or heterologous anti-sera, we incubated the *H. lepturus* venom or rHNC or rLiD1 toxins (10 EC_50_) with each anti-serum (anti-HL, anti-*Loxosceles*, anti-rLiD1) or with control sera (all diluted at 1:50) and mixed with RBCs. Figure 2B indicates that all autologous immune sera completely inhibited the hemolytic effect of *H. lepturus* venom, rHNC, and rLiD1toxins. Anti-HL anti-serum was able to strongly neutralize the hemolytic effect of rLiD1 toxin (about 70%). At the same efficiency rate, we observed that anti-*Loxosceles* and anti-rLiD1sera neutralize the *H. lepturus* venom’ and rHNC’ s hemolytic activities. Normal rabbit and horse sera, used as negative controls, did not affect RBCs hemolysis (data not shown).

### 2.3. Phospholipid Substrates Preference of rHNC and rLiD1

**Figure 3:**
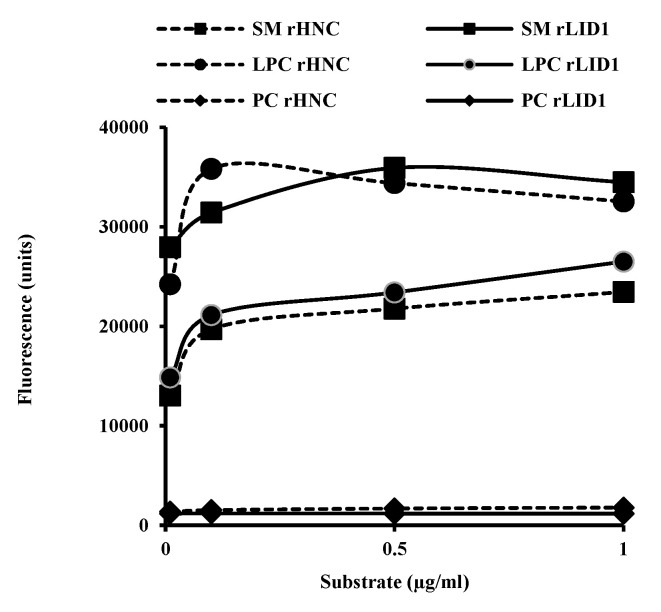
Assessment of rHNC and rLiD1 enzymatic activity. Various concentrations of toxins were incubated with 100 µM of BSA-LPC, SM, and PC for 1 h at 37 °C. The formed choline, oxidized to betaine, was determined fluorometrically. Results are representative of three independent experiments and are expressed as the mean of triplicates ± standard deviation.

In order to compare the enzymatic activity and substrate preference of rHNC and rLiD1, we tested them against the LPC and SM, major substrates previously described for vPLDs. We used PC as a negative control. As expected, both proteins exhibit a double phospholipase activity. Nevertheless, the results illustrated in Figure 3 showed a higher preference of rHNC for LPC than for SM. On the other hand, rLiD1 revealed more pronounced activity against SM rather than LPC. Interestingly, results displayed that the capacity of the rHNC to hydrolyze the LPC is equivalent to that of rLiD1 to hydrolyze the SM and vice-versa.

### 2.4. Transphosphatidylation Potential of rHNC and rLiD1

**Figure 4:**
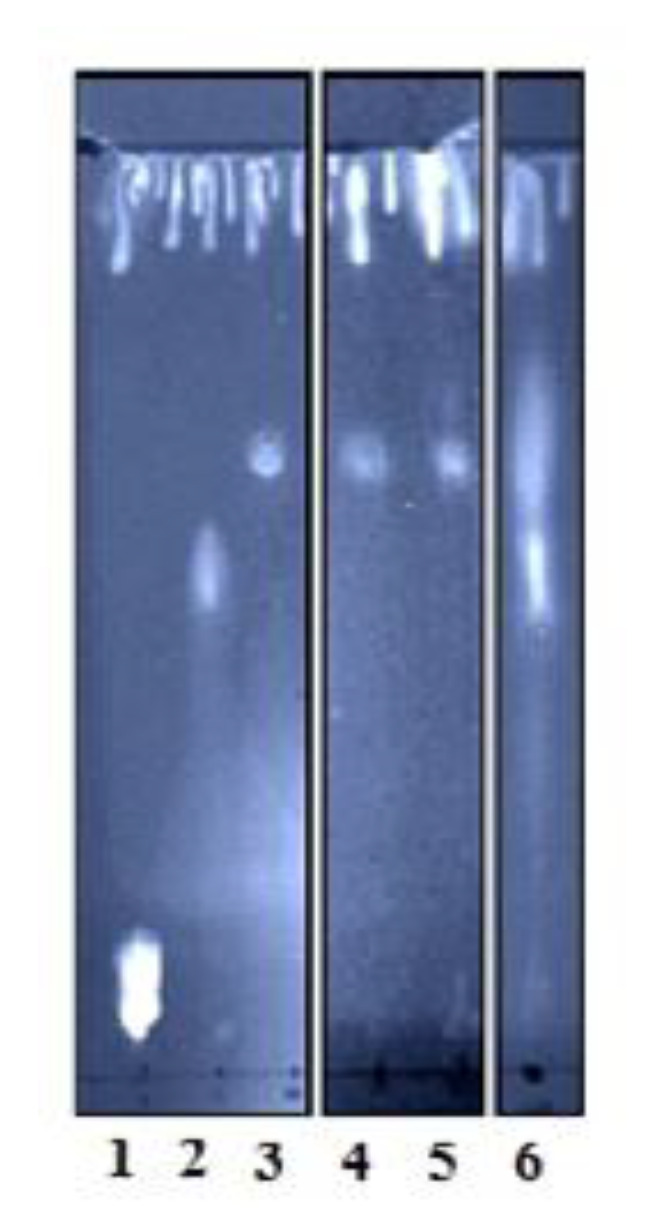
TLC profiles of lipids products of rHNC and rLiD1 enzymatic activity. Primuline detection of phospholipids extracts on silica gel 60 plate after the incubation of each toxin with LPC or LPA substrates and the lipid extraction as described in Materials and Methods. Lane 1: LPC control. Line 2 LPA control. Line 3: cPA control. Line 4: product of rHNC-LPC. Line 5: Product of rHNC-LPA. Line 6: product of rLiD1-LPA.

We aimed herein to further investigate the ability of rHNC, similarly to *Loxosceles* PLDs, to catalyze LPC transphosphatidylation, and to determine whether LPA could be a potential substrate for both toxins. To visualize the hydrolysis products, we select the TLC technique. Results illustrated in Figure 4 showed that HNC endows a transphosphatidylation activity translated by the production of cPA from both substrates LPC and LPA. In contrast, under the used experimental conditions (hydrolysis reaction time), rLiD1 showed a slight activity on LPA substrate (only a smear observed in TLC profile).

### 2.5. Similarities and Differences between rLiD1 and rHNC Revealed by Structural Analysis

To better understand differences in substrate specificity and transphosphatidylase activity between rLiD1 and rHNC, we turned to computational studies. We first built three-dimensional models of the rLiD1 and rHNC sequences, based on the crystallographic structure of a class II PLD from *Loxosceles intermedia* (PDB code 3RLH [12]) (Tables S1 and S2). Typical structural elements of class II LPD, such as the catalytic, variable, flexible and other short loops surrounding the active site cleft, were observed (Figure S1). The disulfide bridges Cys51–Cys57 and Cys53-Cys201 were also present in the models. However, the rHNC model had an extra disulfide bridge between Cys215 and Cys290 at the C-terminal region. For rLiD1, the residue in the 215 position is an alanine and this extra disulfide bridge cannot be formed. Other small differences detected between rLiD1 and rHNC, particularly around the active site (Table S3), resulted in a more negative surface in rLiD1 when compared to rHNC (Figure S2).

### 2.6. Binding and Interaction Predictions of SM and LPC with rLiD1 and rHNC

To investigate the interactions between the toxins and the substrates, SM and LPC were docked into the protein binding sites. The SM and LPC polar groups found similar conformations in both proteins, buried into the pocket (residues His12, Glu32, Asp34, His47, Pro50, Cys51, Asp52, Cys53, Asp91, Lys93, Pro134, Tyr135, Asp164, Ser166, Tyr228, Trp230, Thr199, Cys201) formed by the displacement of the flexible loop towards the catalytic loop (Figures S3 and S4). However, it is interesting to note that the trimethylamine group of the substrates was pointed towards the inside of the pocket in rHNC; thus, interacting with more residues (Glu32, Asp164, and Trp230) than the partially solvent-exposed poses of the substrates found in rLiD1 (see additional analysis in Supplementary material). The aliphatic tail did not find a common conformation between proteins, which might show the flexibility of this group, as expected due to their high solvent exposure.

### 2.7. Dynamic Behavior of rLiD1 and rHNC Structures in Bound and Unbound Simulations

Molecular docking predicted similar binding modes and interactions between SM and LPC when bound to both rHNC and rLiD1, in agreement with the high sequence identity between their binding sites. To investigate the dynamics of these complexes, and search for molecular insights into the experimentally observed difference in their substrate preferences, we carried out MD simulations with and without the ligands.

No major conformation changes were identified from the simulations of rLiD1 and rHNC neither in apo simulations nor in complex with their substrates (Figures S5 and S6). However, a small increase in flexibility was observed in the catalytic loop and the so-called flexible loop in the rHNC systems when compared to rLiD1 (Figure S7A). On the other hand, the loop that comprises Asp91 and Lys93 was more rigid in rHNC than rLiD1 (Figure S7A). These inflexibility differences might be associated with residue substitutions in these regions (Table S3).

### 2.8. Dynamic Behavior of Substrates Bound to rLiD1 and rHNC

Unlike the proteins, the substrates deviated significantly from the initial docking position in the simulations. SM and LPC were flexible in both rLiD1and rHNC binding sites (Figures S8 and S9), mainly due to the mobility of the aliphatic tail. Besides, in the rLiD1 simulations, the initial solvent-exposed trimethylamine group achieved a buried conformation that persisted throughout the simulation. Despite an initial difference in solvent-exposure of the trimethylamine group showed in docking results, among the four analyzed systems the most stable orientation of this group is pointing towards the binding site, where it is stabilized by multiple interactions (see further details in Supplementary material text and Figures S8 and S9). 

### 2.9. rLiD1 and rHNC Intermolecular Interactions with the Substrates

**Figure 5:**
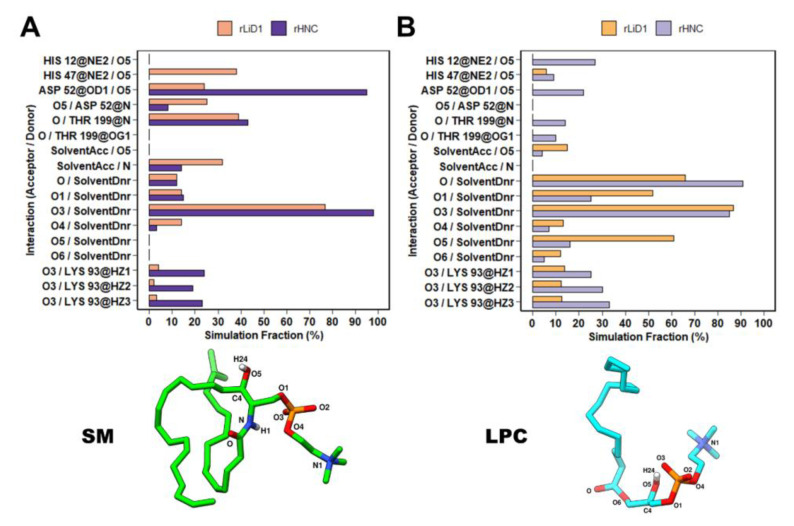
Bond interactions duration throughout simulation time between substrates, proteins, and water molecules. (**A**) Hydrogen bond interactions of SM in the bound simulations of rLiD1 (orange) and rHNC (purple). (**B**) LPC hydrogen bond interactions formed in the rLiD1 (light orange) and rHNC (light purple) simulations. Plots were done with the R program and three-dimensional representations were built with the UCSF Chimera.

**Figure 6:**
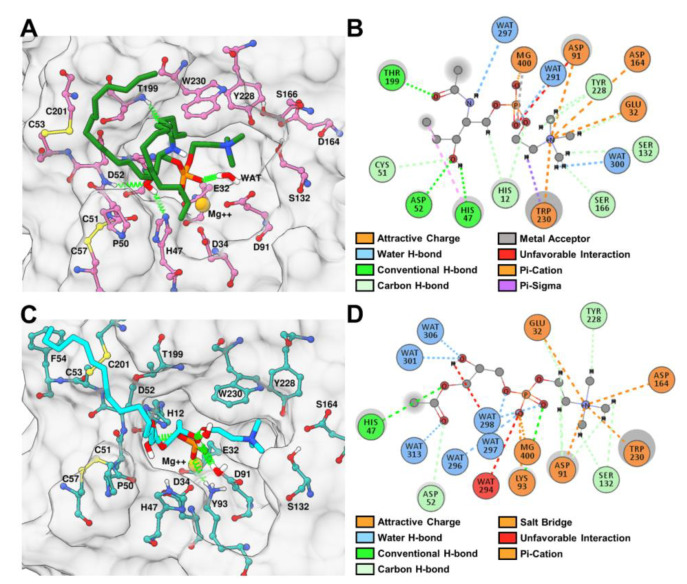
Most sampled MD binding mode and interactions of SM and LPC with rRLiD1 and rHNC, respectively. (**A**) Position of SM (dark green) and the established hydrogen bond interactions (green springs) within the binding site residues of rLiD1. (**B**) 2D interaction map of the polar group of the most sample binding mode of SM in the rLiD1 simulation. (**C**) Position of LPC (cyan) and the formed hydrogen bond interactions (green springs) within the binding site residues of rHNC. (**D**) 2D interaction map of the polar group of the most sample binding mode of LPC in the rHNC simulation. The 3D surface figures were done with UCSF Chimera, while 2D maps were obtained with Discovery Studio Visualizer.

To better understand how rLiD1 and rHNC interact with their substrates, we analyzed the intermolecular hydrogen bond interactions observed during the simulations. The same conserved residues in rLiD1 and rHNC were responsible for these contacts. However, their duration throughout the simulations were different depending on the substrate (Figure 5). For instance, the complex rLiD1-SM maintained hydrogen bond interactions with catalytic His47 (~35% of simulation time), Asp52 (~25% of simulation time, backbone atom N), Thr199 (~39% of simulation time), and water molecules (~32% of simulation time) (Figure 5A). The same hydrogen bond interaction network with His47, Asp52, and Thr199 (~10%, ~22%, and 15% of simulation time, respectively) was found for LPC bound to rHNC (Figure 5B). In addition, the rHNC-LPC simulation achieved hydrogen bond interactions with the other catalytic His12 (~27% simulation time). In the case of rHNC simulations, hydrogen bond interactions with Lys93 were mainly observed. Both His12 and Lys93 are possibly involved in the transphophatidylase activity of toxins [23]. Additionally, as Lys93 is found in the Asp91 loop, this interaction alongside the residue substitutions might be responsible for the increased rigidity of this loop. Moreover, the phosphocholine of both substrates were anchored by carbon-hydrogen bond interactions with Ser132 and Tyr228; and attractive charged interactions with Glu32, Asp91, Asp164, Trp230, and the Mg^2+^ ion (Figure 6A,B). 

### 2.10. Cytotoxicity Effect of HNC and rLiD1 on MDA-MB-231 and MCF7 Cell Lines

**Figure 7:**
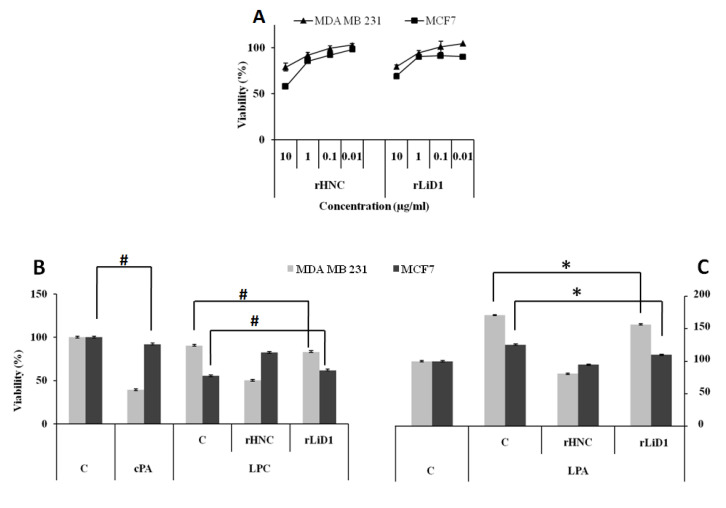
Cytotoxicity and viability of the MDA-MB-231 and MCF7 cell lines under rHNC and rLiD1 treatment with and without exogenous phospholipids. (**A**) MDA-MB-231 and MCF7 cells were incubated for 24 h with different concentrations of rHNC and rLiD1 toxins. The cells were detached from the plates and their numbers were assessed by trypan blue counts. (**B**) The cells were incubated for 24 h with 5 µM LPC alone or added at 1 µg/mL of each toxin. (**C**) The cells were incubated in the presence of cPA (5 µg/mL) or LPA (5 µM) alone or added to the toxins (1 µg/mL). Viability was measured by the MTT reduction assay. Results were expressed as percentage of mean of four independent experiments and shown as mean of triplicate ± SEM, standard errors of the mean. (*) indicate values statistically different from the control (*p* < 0.0005), and (#) indicate no significant difference.

A set of cellular experiments was conducted to investigate and compare the pharmacological effect of the transphosphatidylation activity of rHNC and rLiD1 on lysophospholipid substrates. For this purpose, we selected two breast cancer cell lines with different sensitivity to LPC: MDA-MB-231, a highly aggressive and metastatic breast cancer cell line whose proliferation is unaffected by LPC, and MCF7, a minimally invasive and metastatic breast cancer cell line that is sensitive to LPC. We first studied the cytotoxic effect of both toxins. As shown in Figure 7A, rHNC and rLiD1 do not exhibit severe cytotoxicity for either cell line, except at the highest concentration (10 µg/mL). Therefore, we choose the 1 µg/mL concentration for the following experiments. 

### 2.11. Antiproliferative Potential of HNC and rLiD1 on MDA-MB-231 and MCF7 Cell Lines

The biochemical results described above revealed the ability of rHNC and rLiD1 to produce, to varying degrees, the cPA which is known for its anti-proliferative activity. Based on this fact, we were interested in testing the effect of our proteins on the proliferation of the MDA-MB-231 and MCF7 cell lines in the presence of exogenous LPC. cPA (5 µg/mL) was used as a positive control. As shown in Figure 7B, cPA only inhibited the proliferation of MDA-MB-231 cells (60%), however no activity was observed on MCF7 cells. The LPC (at 5 µM) alone did not affect the rate of MDA-MB-231 proliferation while it was toxic to MCF7 cells (54%). The combination of LPC and rHNC (1 µg/mL) reversed these effects: The proliferation of MDA-MB-231 cells was inhibited by 40% while the toxicity of LPC to MCF7 cells was reversed by 30%. In contrast, no effects were observed under the treatment with the combination of LPC and rLiD1 (1 µg/mL), neither on MDA cell proliferation nor on LPC-induced toxicity on MCF7 cells (*p* > 0.3).

We then raised the question whether the toxins could interfere with the activity of LPA, which is known to be a mitogenic agent for both cell lines. The results shown in Figure 7C demonstrate that rHNC, when added to LPA, strongly inhibits the proliferation of MDA (−50%). Its anti-proliferative activity on MCF7 appears to be less important (−35%). Under the same conditions, rLiD induced inhibition did not exceed 10% (*p* < 0.0005) for both cell lines (Figure 7C). 

## 3. Discussion

In the present study, we have directed our interest toward two venom recombinant PLDs (rHNC and rLiD1) being the main causative agent for all local and systemic effects induced by their consecutive venoms of *H. lepturus* scorpion and *brown intermedia* spider. Since both toxins have similar in vivo activities and sequence similarity (48%), we argued if they have similar enzymatic and antigenic properties.

For antigenicity, the ELISA results showed a clear cross-reactivity for the two anti-crude venom sera (anti-HL, anti-*Loxosceles*) against rHNC and rLiD1. However, the reactivity of the anti-*Loxosceles* antiserum against *H. lepturus* venom is lower than the one against rHNC (Figure 1A). This can be explained by the different antigenic components present in both venoms. Indeed, with the exception of dermonecrotic toxins, no other homologous proteins have been described between *Loxosceles* and *Hemiscorpius.* In addition, preincubation of anti-*Loxosceles* with rLiD1 completely suppresses its reactivity with *H. lepturus* venom. On the other hand, competition ELISA assays revealed additional observations. Anti-HL sera preincubated with rHNC toxin slightly decrease reactivity against *H. lepturus* venom (Figure 1B). This finding indicates that rHNC is not the most immunogenic toxin in the venom. Transcriptomic analysis revealed that only 19% of the venom is composed by enzymes, which besides HNC contains phospholipase A2 enzymes, metalloproteinases, and serine proteinases, appearing to be more immunogenic than rHNC [21]. Therefore, although anti-HL neutralize the pathophysiological effect of *H. lepturus* venom [20], it seems that most anti-HL antibodies are not directed against PLD proteins. Another interesting finding is that the anti-HL serum recognizes rLiD1 better than anti-*Loxosceles* recognizes rHNC. This suggests that other isoforms present in *H. lepturus* venom are antigenically closer to rLiD1 than rHNC. Regarding this point, the transcriptomic study of *H. lepturus* showed the presence of three other PLD isoforms [21]. In this context, the reactivity of the anti-rLiD1 antiserum against rHNC was only reduced, but not suppressed, by rLiD1 (Figure 1C). Sequences alignment (Figure S10) indeed proclaims a difference in the epitopes of both toxins, since among the three epitopes already identified for rLiD1, only two of them are similar on the rHNC toxin [24]. In addition, the toxins can also have different conformational epitopes.

Referring to the literature, including our previous studies, it has been demonstrated that anti-venom sera or anti-dermonecrotic toxins for both venoms and toxins neutralize the pathophysiological effects of venoms in vitro and in vivo [16,20,25]. We tested here the ability of the sera used in this study to control complement-dependent erythrocyte hemolysis, known to be one of the major effects of envenomation. Our results displayed the capacity of anti-crude venoms sera as well as the one directed against rLiD1 for inhibiting the toxin induced hemolysis with a rate reaching 70%. In addition, anti-rLiD1 antibodies were as potent as anti-*Loxosceles* antibodies in neutralizing the hemolysis induced by *H. lepturus* venom. These results confirm that PLD toxins for both venoms share epitopes associated with neutralizing antibodies.

As we demonstrated that rHNC and rLiD1 were capable of generating cross-reactivity, cross-neutralizing antibodies as well as their sequence similarity, prompted us to further explore their enzymatic characteristics. The enzymatic activity of the rLiD and rHNC toxins carried by the fluorometric choline release assay confirmed the substrate preference of each toxin. We showed that rLiD1 and rHNC are able to process both SM and LPC, while SM and LPC are the preferential substrates for rLiD and rHNC respectively. Despite their different substrate preferences, the two enzymes appear to have the same enzymatic efficiency since the enzyme curves for rLiD1 processing SM and that for rHNC processing LPC are similar. Recently, Lajoie et al., 2013 [10] showed by ^31^P NMR and mass spectrometry that spider venom toxins have transphosphatidylation activity and catalyze the cyclization of lysophospholipid substrates. Using the TLC phospholipid revelation, we have clearly demonstrated the exclusive transphosphatidylation activity of the rHNC protein. The transphosphatidylation characteristic attributed to PLD spiders can be extended to the *H. lepturus* venom accordingly.

Due to the high similarity among SMasesD active sites, the determinants of substrate specificity in this enzyme family are still not well understood. Our structural analysis showed similar binding modes of SM and LPC in both proteins. However, when we considered the dynamics of these systems and analyzed the frequency of interactions between proteins and substrates, we observed similar interactions networks for the complexes rLiD1-SM and rHNC-LPC throughout the simulations. The higher frequency of hydrogen bond interactions with His12 and His47 (catalytic residues), Asp52 (found in the catalytic loop), and Thr199 (found in the flexible loop) in complexes of each toxin with their preferential substrate suggests their importance for substrate cleavage and preference. Additionally, throughout our MD simulations the phosphocholine of both substrates interacted with Glu32, Asp91, Ser132, Asp164, Try228, Trp230, and the Mg^2+^ ion. Our results are in agreement with previous studies that described that all conserved residues (Glu32, His47, Asp91, Lys93, Tyr228, and Trp230) affect substrate affinity, enzymatic activity, and ion coordination [26,27]. 

In addition, analysis of hydrogen bonds between LPC and the toxins rLiD1 and rHNC also provide a possible explanation for the higher transphophatidylase activity observed for rHNC. In a study with toxins from *Sicariid* spiders, Lajoie et al., 2015 [23] proposed two mechanisms for the formation of cPA, through a nucleophilic attack from O5 to the phosphodiester (see numbering in Figure 5B). In this process, deprotonation of O5 is important to provide an efficient nucleophile, and their docking results suggested two LPC binding modes, which would favor either His12 or His47 as the base to abstract the proton from O5 [23]. In agreement with these mechanisms, and our finding that rHNC has higher transphophatidylase activity than rLiD1, our MD simulations revealed a higher frequency of hydrogen bonds between LPC O5 and both rHNC His12 and His47, when compared to the rLiD1-LPC system. The observed difference was more significant for His12, found to interact with O5 during 27% of the time in the rHNC simulation, while this interaction was not observed with rLiD1. 

All the work undertaken so far to understand the pathophysiology of *Loxosceles* spiders or *H. lepturus* scorpion venoms has been aimed at studying the signaling pathways of the two lipid mediators, LPA and 1-phosphate ceramide, which would be formed by the enzymatic reaction of vPLD toxins. However, recent discoveries by the Lajoie team [10], similar to our current study, have shown that these toxins generate exclusively cyclic phosphate such as cPA from the LPC substrate. In fact, under physiological conditions, LPA is derived from the enzymatic activity of a serum protein, autotaxin (ATX) on LPC [28]. It is present in several biological fluids, including peripheral blood [29]. LPA signaling is specific and mediated by at least five G protein coupled receptors as (LPA_1-5_) [30]. Although LPA and cPA are structurally similar, they have distinct and opposing biological activities (see below). It is therefore quite plausible to consider LPA as a potential substrate for vPLD toxins. To our surprise, the results showed that both enzymes catalyze the intra-molecular transphosphatidylation of LPA. rHNC showed rapid and complete turnover, while rLiD1 showed only partial activity, most likely due to its preference for the ceramide-based substrate and its limited enzymatic efficacy for lysophospholipids. In addition, according to Lajoie and collaborators [23], spider PLDs have activity against lysophospholipids with positively charged head groups (choline and ethanolamine) but show no detectable activity against neutral/zwitterionic head groups (glycerol and serine). We report here, for the first time, that LPA, a zwitterionic lysomonoester without head groups, could be a molecular target for these toxins.

To extend our ongoing study to cellular systems, we have explored the effects of both toxins on two breast cancer cells in the presence of exogenous LPC and LPA. LPC has been shown to suppress cell proliferation and to exhibit cytotoxicity to several cell types [31], while LPA has multiple actions that promote the growth, migration, invasion, and survival of cancer cells [32]. Unlike LPA, cPA has shown antiproliferative activity on fibroblasts [33] and inhibitory activity on cancer cell invasion and metastasis [34]. In this study, we treated two commonly used human breast cancer cell lines: MDA-MB-231, whose proliferation is unaffected by LPC, and MCF7 sensitive to LPC. Our results revealed that, unlike the LPC-rLiD1 combination, the LPC-rHNC one significantly reduced the cytotoxicity of LPC on the MCF7 cell line. These results are consistent with the substrate preference of each enzyme. Therefore, rHNC, through its enzymatic activity, reduced the concentration of LPC, thereby reversing its cytotoxic effect on MCF7 cells. On the other hand, while the generated cPA seems to affect the proliferation of MDA cells, it does not have any action on MCF7 cells. Previous studies have shown that cPA interacts directly with cell cycle regulatory proteins, resulting in the arrest of cell cycle progression and inhibition of cell proliferation by caspase-dependent apoptotic pathways [35]. The MCF7 cell line, known not to express caspase-3 due to a deletion in the *casp-3* gene [36], was therefore able to escape cPA action. In contrast, by transforming LPC into cPA, the rHNC toxin decreased the viability of MDA cells. The results obtained in the presence of rHNC-LPA confirm these observations, since the anti-proliferative effect observed in MCF7 cells is much weaker than that observed in MDA cells and appears to be due solely to a decrease in LPA concentration. In addition, exogenous cPA affected only the viability of MDA cells. It appears that rHNC, through its lipolytic product, acts as an apoptotic molecule. Experiments are underway to further unravel the molecular mechanisms triggered by rHNC to perform its anti-proliferative and apoptotic activities. Otherwise, our data on rLiD1-LPA strongly support the low activity of this toxin on lysophospholipid substrates comparatively to rHNC according to the dose and incubation time used in this study, although LPA appears to be a better substrate than LPC, since a 10% inhibition was observed in both cell lines. The Tambourgi group has well established the apoptotic activity of the spider toxin in HaCaT keratinocytes. They have used, however, a toxin concentration of 15 μg/mL (15 times higher than ours) and an incubation time three times higher [37]. As observed in erythrocytes, rHNC likewise appears to be more active than rLid1 toxin on nucleated cells. Moreover, all undertaken spider toxins studies characterized signaling pathways downstream of toxin binding, without demonstrating a direct correlation with cyclic ceramide phosphate—its main enzymatic product [38]. Future research on the biological effects of cyclic phospholipids could shed more light on the molecular basis of the envenomation of the scorpion *H. lepturus* as well as on loxoscelism. 

## 4. Conclusions

In this study we observed that anti-venoms against *H. lepturus* and *Loxosceles* cross-react with both major respective venoms toxins (rHNC and rLiD1), indicating a cross antigenic property of these dermonecrotic toxins. This finding was confirmed by the cross-reactivity of anti-rLiD anti-serum with *H. lepturus* venom and rHNC. We also observed a higher hemolytic activity of rHNC compared to rLiD1 and a partial cross-neutralization of the hemolytic activities induced by the recombinant toxins. This study showed a substrate preference of rHNC for LPC and rLiD1 for SM, most likely related to the network of hydrogen bond interactions between the enzymes and the substrates. Additionally, we have demonstrated that cPA is the final product of the reaction for rHNC as indicated by enzymatic and in vitro proliferation studies.

## 5. Materials and Methods 

### 5.1. Toxins and Venom

Recombinant dermonecrotic PLD from *H. lepturus* (HL) venom (referred to as rHNC in this work), was produced by Torabi and collaborators [22]. rLiD1was expressed and purified according to Oliveira et al., 2015 [15]. *H. lepturus* scorpions were collected from Khuzestan by the veterinarian service of the Razi Vaccine Development and Serum Research Institute of Iran. Venom was obtained by mild electrical stimulation of the telsons, extracted with distilled water, and centrifuged at 10,000× *g* for 15 min. Aliquots of collected supernatant were stored at −20 °C until use.

### 5.2. Anti-Venoms and Anti-Toxins 

The *Loxosceles* anti-venom was provided by Dr. JoãoMinozzo from the Centro de Produção e Pesquisa de Imunobiológicos (CPPI) in the state of Paraná-Brazil. Anti-Loxosceles anti-venom was produced by horse immunization against *L. intermedia, L. gaucho* and *L. laeta* venoms. Anti-rLiD1 antibodies were purified from anti-*Loxosceles* anti-venom in a single step by immunoaffinity chromatography using rLiD1 immobilized on CNBr-Sepharose column. Whole venom *H. lepturus* was used as an immunogen to produce anti-venom antibodies [20].

### 5.3. Immunoassays

MaxiSorp plates were coated overnight at 4 °C with 100 µL of a 2.5 μg/mL solution of rLiD1, rHNC, or *H. lepturus* venom in 20 mM sodium bicarbonate buffer, pH 9.6. Plates were washed with saline containing 0.05% Tween 20 and blocked for 1 h at room temperature with PBS containing 0.25% casein. Plates were incubated with eight different dilutions of each one of the antibodies (anti-HL, anti-*Loxosceles* and anti-rLiD1): (1:100, 1:500, 1:1000, 1:5000, 1:10,000, 1:50,000, 1:100,000, 1:250,000). For competition ELISA, the different Antigens (10^−6^ M) were incubated for 30 min at 37 °C with each dilution of each antibody, before adding to the plates. After 1 h at 37 °C the plates were washed and incubated with either horseradish-peroxidase (HRP)-labeled rabbit anti-horse or goat anti-rabbit (1:5000) (Southern Biotechnology, Birmingham, AL, USA) for 1 h at 37 °C. Plates were washed, and incubated in the dark with H_2_O_2_ in the presence of ortho phenylenediamine (OPD, Sigma, Saint Louis, MO, USA) in sodium citrate buffer; pH 5.0 for 20 min. The reaction was stopped with 20 μL of 2 N H_2_SO_4_. Optical density was measured using an automatic ELISA reader at 492 nm. All measurements were made in triplicate. The antibody titer was considered as the highest dilution of serum capable of resulting in a reading greater than 1.0.

### 5.4. Complement-Dependent Hemolytic Activity

Fresh red blood cells (RBCs) were obtained as previously described by Borchani and co-authors (2011b) [20]. To determine the concentrations causing 50% RBCs hemolysis (EC_50_) with each component, human erythrocytes were washed three times with VBS^2+^ and resuspended in the same buffer at 2%. The cells were sensitized with the proteins (HL crude venom, rHNC or rLiD1; concentrations ranging between 10 ng/mL and 400 ng/mL) for 30 min at 37 °C. Control samples were incubated with VBS^2+^ buffer alone. The sensitized erythrocytes were then washed, resuspended to the original volume and mixed with 100 µL of autologous serum (hemolytic step). After incubation for 1 h at 37 °C, non lysed cells were discarded. The light absorbance of the released hemoglobin was measured at 405 nm. Total cell lysis was evaluated by incubation erythrocytes with distilled water. 

Hemolysis percentages were given by the equation (OD e−OD 0)/(OD 100−OD 0) × 100; where OD e, OD 0, and OD 100 correspond to optical density respectively to the toxin, spontaneous and total lysis of erythrocytes. Results are representative of three independent experiments expressed as the mean of duplicates ± standard deviation.

### 5.5. Cross Inhibition of Hemolysis

In order to study the cross neutralization hemolytic activity of spider and scorpion venoms by the different anti-sera (anti-HL, anti-*Loxosceles* and anti-rLiD1), we analyzed the inhibition of RBCs’ lysis induced by *H. lepturus* whole venom, or by rHNC and rLiD1 toxins. Before the sensitization step of RBCs, preparations containing 10 EC_50_ quantity of each hemolytic agent were mixed (*v*/*v*) during 30 min at 37 °C with the different anti-sera diluted at 1:50. Control mixtures were incubated with normal rabbit or horse serum (1:50). The sensitization and hemolysis steps are the same as those previously described. Tests were performed in duplicates. ODs means and standard deviations (SD) were determined from three independent experiments. The percentage of neutralization of hemolysis is expressed as follows: (percentage of hemolysis in the presence of antiserum/percentage of hemolysis in the presence of normal serum) × 100.

### 5.6. Enzymatic Activities

#### 5.6.1. PLD Activity of rHNC and rLiD1: Choline Release Assay

In order to check if both toxins bear SMaseD and lysoPLD activities, we analyzed their capacity to hydrolyze BSA-LPC and SM Substrates. Phosphatidylcholine (PC) was tested in parallel as a negative control. The PLD enzymatic activity was assessed by measuring the choline liberated from lipid substrates, using a fluorometric assay [39]. In the standard assay, the substrate was diluted in 200 mL of HBS buffer. SM and PC substrates (both at 100 µM) were applied as liposomes, whereas LPC was complexed to free fatty acid—BSA (5 mg/mL). After toxins addition (0.01, 0.1, 0.5, 1 µg/mL), the reaction was left to proceed for 1 h at 37 °C with gentle shaking. By adding 50 µl of a second assay mixture, consisting of 1 U/mL choline oxidase, 0.6 U/mL horseradish peroxidase and 200 µM HPPA in HBS, the liberated free choline was oxidized (in 1 h) to betaine, and the H_2_O_2_ concomitantly generated was determined by fluorimetry. Calibration curves were built using H_2_O_2_ as a standard fluorescence of the oxidized substrate, which was measured by fluorimetry at em¼ 405 nm and ex ¼ 320 nm, using 96- well microtiter plates, in a spectrofluorometer (Perkin–Elmer, Waltham, MA, USA). Results are representative of three independent experiments and are expressed as the mean of triplicates ± standard deviation.

#### 5.6.2. Thin Layer Chromatography: Detection of Lipids Products from rHNC and rLiD1 Toxins Enzymatic Activity

LPC and LPA phospholipids were complexed to free fatty acid, BSA (5 mg/mL), at room temperature overnight under shaking. Liposomes were then sonicated for 60 min then added to the reaction mixture contained rHNC or rLID1 at 1 µg in CaCl_2_ and MgCl_2_ (1 mM) HEPES buffer. The reaction was conducted at 37 °C under shaking for 45 min and stopped by adding 0.35 volume of 0.1 M citric acid. Lipid extraction was then carried out using 5.4 volumes of Chloroform:Methanol (2:1). After vigorous stirring, the mixtures were centrifuged at 1400× *g* for 5 min. The upper phases were reextracted once more following the same process whereas the lower phases were dried under N_2_ gas stream. The extracted lipids were resuspended in a small amount of Chloroform:Methanol (2:1) and spotted on a silica gel 60 thin layer chromatography plate. The plates were developed with a solvent system consisting of Chloroform:Methanol:Acetic acid:1% sodium disulfide aqueous solution (100:40:12:5). Lipids were revealed by spraying plates with Primuline staining (5 mg in 100 mL acetone/water 80:20). Lipids appear as yellow spots under UV 340 nm.

### 5.7. Structural Studies

#### 5.7.1. Comparative Modeling

After obtaining the rLiD1 and rHNC sequences, the basic local alignment search tool (BLAST) was used to identify suitable templates in the Protein Data Bank (PDB) [40]. For both target sequences, we selected a class II PLD from *L. intermedia* (PDB code 3RLH [12]), with 1.72 Å resolution, as the template. Template and target sequences were aligned using the PSI-Coffee mode of the T-Coffee program [41]. Based on the alignment, N-terminal residues of the target sequences without coverage were removed from modeling.

Modeller version 9.22 [42] was used to create 500 homology models with the standard auto model for each sequence and optimize them via the variable target function method (VTFM) with up to 300 interactions. Further optimization was conducted with the molecular dynamics (MD) module in the slow level mode. The full cycle was repeated twice to produce an optimized conformation of the model. A disulfide bond patch was included in the Modeller script to incorporate an extra disulfide between Cys215 and Cys290 (C-terminal region) in the rHNC sequence, absent in the template. The Mg^2+^ ion of the template structure was also modeled into the structures.

The modeled structures were chosen by their discrete optimized protein energy (DOPE) score and refined using the 3Drefine server [43]. Initial and optimized models were assessed by the QMean server [44], while the ERRAT [45], Ramachandran plot, and Verify3D [46] steps were calculated on the Structural Analysis and Verification Server (SAVES) of UCLA-DOE Lab for stereochemical and energetic analysis. 

#### 5.7.2. Molecular Docking

Molecular docking runs were performed using the AutoDock4.0 program [47]. The AutoDock tools suite was used to prepare the proteins and ligands. During the preparation, polar hydrogens were incorporated into the rLiD1 and rHNC models, and Kollman United atom charges and atomic salvation parameters were assigned. SM and LPC structures were extracted from the Pub chem database [48], and Gasteiger atomic charges were appointed.

Electron affinity and electrostatic potential were calculated with the Autogrid program [47]. The grid map comprised 85 × 100 × 120 points, with a grid spacing of 0.375 Åusing distance-dependent dielectric constants. Molecular docking was performed using 200 runs of the GA-LS method with a maximum number of energy evaluations of 27,000,000 for each run. After docking, all the generated structures were assigned to clusters based on a tolerance of 2.0Åall-atom root-mean-square deviation (RMSD) from the lowest-energy structure. Specific interactions between the proteins and the possible binding modes were analyzed using UCSF Chimera [49] and Discovery Studio Visualizer [50].

#### 5.7.3. Molecular Dynamics

MD simulations were carried out using AMBER 16.0 [51] with the ff14SB force field [52] to represent protein interactions. Bonded, electrostatic, and Lennard-Jones parameters for SM and LPC ligands were acquired using the generalized amber force field (GAFF) [53]. AM1-BCC atomic partial charges [54] were added using the ANTECHAMBER program [55]. Electrostatic interactions were treated using the Particle-Mesh Ewald (PME) algorithm with a cut-off of 10 Å. Each system was simulated in an octahedral box filled with TIP3P water molecules, considering 11 Å from the outermost protein atoms in all Cartesian directions. Protonation states of titratable residues were assigned using PDB2PQR software [56]. All systems were neutralized by adding 2Cl^−^ counterions.

Minimization was performed in two consecutive procedures of 2000 steps (1000 steepest descent followed by 1000 conjugate-gradient steps). In the first procedure, all heavy atoms were harmonically restrained with a force constant of 5 kcal mol^−1^ Å^−2^. In the second procedure, position restraints were lifted. Then, initial atomic velocities were assigned using a Maxwell-Boltzmann distribution corresponding to an initial temperature of 20 K and the systems were gradually heated to 310 K over 500 picoseconds applying the Langevin thermostat. During this stage, all heavy atoms were harmonically restrained with a force constant of 10 kcal mol^−1^ Å^−2^. Systems were subsequently equilibrated during nine successive 250 ps equilibration simulations where position restraints gradually approached zero. After this period, the systems were simulated with no restraints at 310 K in the Gibbs ensemble with a pressure of 1 atm. Production simulations were performed for rLiD1 and rHNC models with no ligand and complexed with SM and LPC for 100 ns each.

Trajectories analysis was performed using cpptraj, part of the Amber Tools version 18.0 [57]. Hydrogen bond analysis was also performed with cpptraj to track interactions between the proteins and ligands. VMD [58] was used to visualize the trajectories, calculate the root-mean-square deviation (RMSD) and root-mean-square fluctuation (RMSF) of each simulation. Since N-terminal and C-terminal residues are very flexible, they were not considered in the RMSD calculations. All plots were illustrated with program R [59].

### 5.8. Cellular Studies

#### 5.8.1. Cell Culture

The human breast epithelial cell lines MCF7, an estrogen receptor-positive cell line derived from an in-situ carcinoma, and MDA-MB-231, an estrogen receptor-negative cell line derived from a metastatic carcinoma, were used in our current study. Both cell lines were grown in DMEM medium supplemented with 10% heat-inactivated fetal bovine serum, 2 mM L-glutamine and 1% penicillin/streptomycin. Cells were routinely maintained in a humidified atmosphere with 5% CO_2_ at 37 °C. Treatments cells were counted then plated. After 24 h, when cultures reached 70 to 80% of confluency, the medium was replaced with a medium without FBS and treatments were added.

#### 5.8.2. Viability

In the order to consider the cytotoxic effect of rHNC and rLiD1, MDA-MB-231 and MCF7 cells were seeded into 96 well plate at 10,000 cells/mL incubated at 37 °C and 5% CO_2_ for 24 h and then treated with various concentrations of the two toxins (0.01, 0.1, 1, 10 µg/mL) and further incubated for 24 h. Viability was assessed by trypan blue staining. Results were expressed as percentage of mean of four independent experiments of the viable cell number in treated cells relative to untreated cells.

#### 5.8.3. Anti-Proliferative Potential Determination of rHNC and rLiD1

Cell viability was measured by MTT reduction via mitochondrial oxidation of living cells. Cells were treated with standards LPC or LPA (5 µM) in order to determine their own toxicity on cells and get a relative comparison for the toxins’ proliferative potential. One µg rHNC or 1 µg rLID1 was added in the presence of LPC or LPA (5 µM). Untreated cells were also maintained in culture for 24 h. MTT reagent was added to treated cells at a final concentration of 0.5 mg/mL and cultures were continued at 37 °C and 5% CO_2_ for 3 h. Dimethyl sulfoxide (DMSO) was added to dissolve the blue formazan crystals and the absorption was measured at 540 nm using a microplate reader. Cell survival was calculated in percentage as follows: Cell viability (%) = OD (treated cells)/OD (untreated cells) × 100. Results were expressed as percentage of mean of four independent experiments and shown as mean of triplicate ± SEM, standard errors of the mean. The unpaired Student’s *t*-test was used to determine differences between mean values. *p*-values of less than 0.0005 were considered significant.

## Supplementary Materials

The following are available online at https://www.mdpi.com/2072-6651/12/10/631/s1, Figure S1: Ribbon representation of the rLiD1 (A) and rHNC (B) modeled structures. Figure S2: Electrostatic surface charge distribution from red (−2 kV) to blue (+2 kV) of rLiD1(A) and rHNC (B). Figure S3: Predicted binding modes for SM in rLiD1 (A and B) and rHNC (C and D) from molecular docking, Figure S4: Predicted binding modes for LPC in rLiD1 (A) and rHNC (C) from molecular docking. Figure S5: Average Cα-RMSD plots for all three simulations (apo, SM and LPC bound) of rLID1 and rHNC. Figure S6: Superimposed representative structures obtained from clustering the simulation trajectories of each rLiD1 and rHNC system. Figure S7: Per-residue fluctuation in apo and bound rLiD1 and rLiD1 simulations, using the unbound rLiD1 simulation as reference (∆RMSF = RMSF_simulation_ − RMSF_rLiD1apo_). Figure S8: SM deviation throughout the rLiD1 and rHNC bound simulations. Representative structures of the most sampled ligand conformations, calculated by clustering the trajectories, are displayed compared to docking pose. Figure S9: LPC deviation throughout the rLiD1 and rHNC bound simulations. Representative structures of the most sampled ligand conformations, calculated by clustering the trajectories, are displayed compared to docking pose. Figure S10: Pairwise Sequence alignment between rLiD1(UniProtKB/Swiss-Prot: P0CE81.1) and rHNC (UniProtKB/Swiss-Prot: A0A1L4BJ98) using Needleman-Wunsch algorithm. Table S1: BLASTP analysis of the target rLiD1 and rHNC and the selected template structure. Table S2: Evaluation of rLiD1 and rHNC models after the optimization procedure. Table S3: Differences in the binding sites between rLiD1 and rHNC structures.
