# Supplementary material for: Antigenic and Substrate Preference Differences between Scorpion and Spider Dermonecrotic Toxins, a Comparative Investigation
Source: Toxins (Basel). 2020 Oct 1;12(10):631. doi: 10.3390/toxins12100631 (PMC7601583; doi:10.3390/toxins12100631)
Supplement: Supplementary file 1 [file toxins-12-00631-s001.pdf]

Supplementary Materials: Antigenic and Substrate Preference Differences between Scorpion and Spider Dermonecrotic Toxins, a Comparative Investigation

Ramla Ben Yekhlef, Liza Felicori, Lucianna Helene Santos, Camila F. B. Oliveira, Raoudha Fadhloun, Elham Torabi, Delavar Shahbazzadeh, Kamran Pooshang Bagheri, Rafaela Salgado Ferreira and Lamia Borchani

Similarities and Differences between rLiD1 and rHNC Revealed by Structural Analysis

Aiming to analyze the structural differences between these toxins, we first built 3D models of the recombinant form of *L. intermedia* dermonecrotic (rLiD1) and recombinant Heminecrolysin (rHNC) sequences based on the crystallographic structure of a class II PLD from *Loxosceles intermedia* (PDB code 3RLH [1]) (Table S1). After choosing and validating the models (Table S2), we analyzed their structural features. The catalytic, variable, flexible, and other short loops surrounding the active site cleft were conserved (Figure S1). For instance, the two catalytic residues [2], His12 and His47, and the residues Glu32, Asp34, Asp91, known to coordinate the Mg^{2+} [3] were observed and structurally aligned in both models. The associated disulfide bridges of class II PLD, Cys51-Cys57, and Cys53-Cys201, were conserved in both rLiD1 and rHNC. An extra disulfide bridge between Cys215 and Cys290 at the C-terminal region was found in the rHNC model. Other important substrate binding residues, such as Lys93, Tyr228, and Trp230 were also preserved between sequences [3].

Small differences between rLiD1 and rHNCwere found due to various residue substitutions distributed along the proteins' sequences. Among these differences, 25 are close to the binding site (Table S3), impacting the charge distribution and surface shape of the catalytic interface (Figure S2), which could affect substrate affinity [2]. Since specific inter- and intra- molecule contacts contribute to substrate binding, residue substitutions may affect important interaction networks. Therefore, to investigate the interactions between the proteins and the substrates, SM and LPC, molecular docking was carried out.

Table S1. BLASTP analysis of the target rLiD1 and rHNC and the selected template structure.

Target	Template	Identity (%)	Similarity (%)	Cover (%)	Gaps (%)	Max Score	E-Values
rLiD1	3RHL	88.9	89.0	99.0	8.0	579	0.0
rHNC	3RHL	46.2	69.0	94.0	1.0	275	1×10^{-91}

Table S2. Evaluation of rLiD1 and rHNCmodels after the optimization procedure.

Models	DOPE Score[a]	ERRAT[b]	Verify3D[c]	QMean[d]	Z-score[e]	Ramachandran Plot[f]			
						R1	R2	R3	R4
rLiD1	−33621	98.2	98.2%	−0.9	−9.1	99.3%	0.0%	0.0%	0.4%
rHNC	−34377	89.3	94.5%	0.1	−7.9	98.3%	1.4%	0.0%	0.3%

[a]More negative DOPE (DiscreteOptimizedProteinEnergy) scores values tend to correlate with more native-like models. DOPE was generated through many interactions by MODELLER script [4]. [b]A ERRAT [5] score over 80 displays that only a few residues have an elevated error function (error > 95% confidence limit) when compared to similar experimental structures. ERRAT was calculated by theSAVES server of UCLA-DOE Lab [6]. [c]Verify 3D [7] result over 80% indicates that the amino acids have compatibility between the 3D model and the amino acid sequence. Verify 3D was calculated by the SAVES server of UCLA-DOE Lab [6]. [d]QMean [8] scores values close to zero indicate that the geometrical properties (both global and local) are similar to what one would expect from experimental structures of the same size. QMean values were obtained from Swiss model expasy.org [9]. [e]Z-scoreis used to test if the knowledge-based potentials could recognize a native fold, found in experimental structures, from other alternatives. The Z-score for these structures had to be within

the acceptable range of −12 to 12. Z-score was calculated by the ProSA Web server [10]. [f]R1: Residues in most favored regions; R2: Residues in additional allowed regions; R3: Residues in generously allowed regions; R4: Residues in disallowed regions.

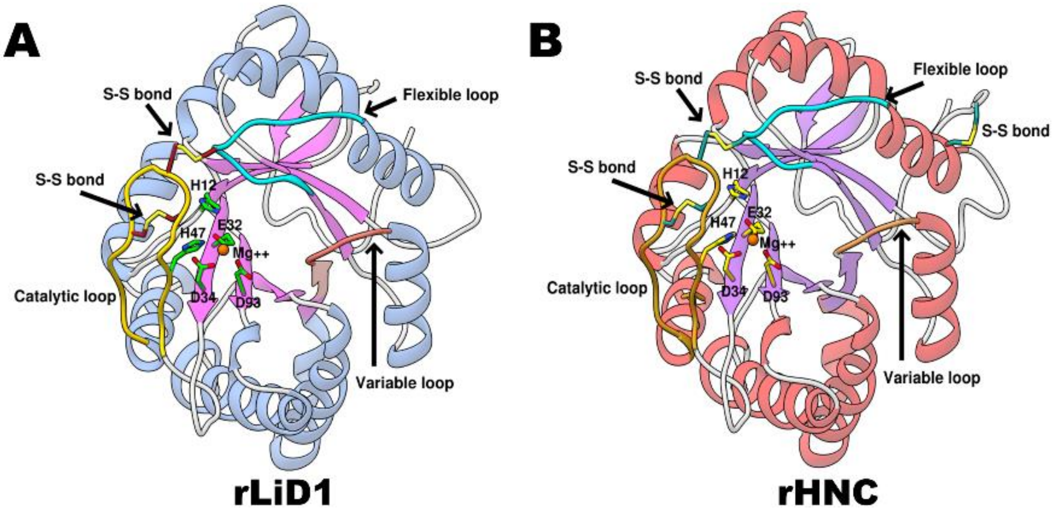

Figure S1. Ribbon representation of the rLiD1 (**A**) and rHNC (**B**) modeled structures. **A**) In the rLiD1 structure, the catalytic histidines (H12 and H47) and the three residues (E32, D34, and D93) that coordinate the metal ion (orange sphere), conserved in Class II phospholipase D (PLD), are shown as sticks with carbon atoms in green. The catalytic, flexible, and variable loops are colored yellow, cyan, and salmon, respectively. The two disulfide bridges, uniquely present in Class II members, are highlighted red. **B**) In the rHNC structure, the catalytic histidines (H12 and H47) and the three residues (E32, D34, and D93) that coordinate the metal ion (orange sphere) are shown as sticks with carbon atoms in yellow. The catalytic, flexible, and variable loops are colored gold, cyan, and salmon, respectively. The two disulfide bridges, uniquely present in Class II members, and one additional disulfide bridge found in the rHNC sequence are highlighted blue. Three dimensional representations were built with the UCSF Chimera [11].

Table S3. Differences in the binding sites between rLiD1 and rHNC structures.

Position	rLiD1	rHNC	Protein Region
10	Met	Ile	Near His12, Glu32 and Asp34
31	Ile	Leu	
33	Thr	Ala	
36	Ser	Thr	
44	Tyr	Trp	Catalytic Loop
49	Ile	Thr	
54	Gly	Phe	
56	Asn	Asp	
58	Lys	Leu	
59	Lys	Arg	
60	Tyr	Trp	
88	Val	Leu	Near Asp91
89	Val	Met	
90	Phe	Leu	
94	Thr	Ile	
95	Gly	Ser	
96	Ser	Lys	
98	Tyr	Ser	
101	Gln	Ala	
163	His	Trp	Variable Loop

165	Phe	Ile	
174	Asp	Glu	
203	Phe	Thr	
205	Gly	Ser	Flexible Loop
207	Leu	His	

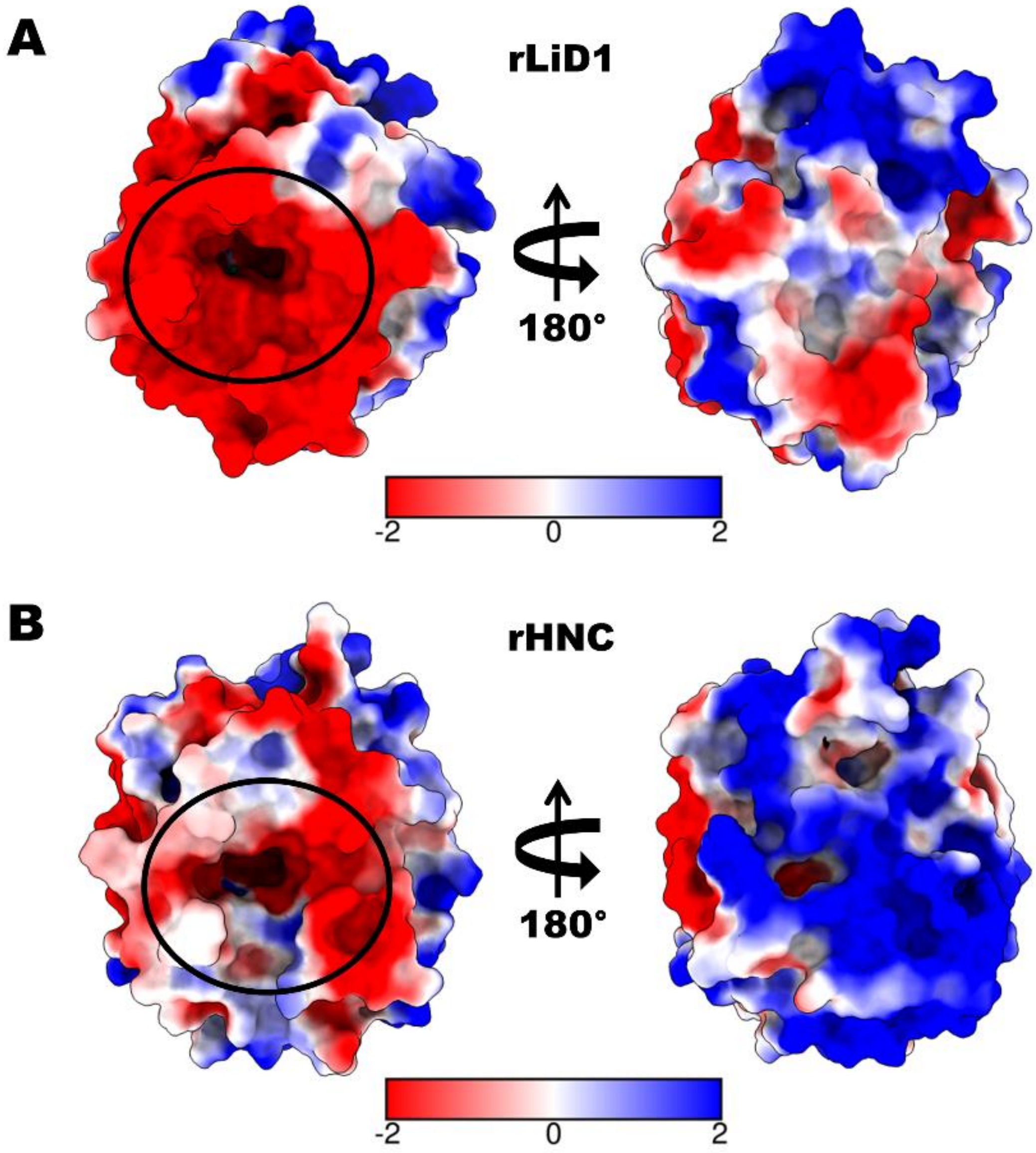

Figure S2. Electrostatic surface charge distribution from red (−2 kV) to blue (+2kV) of rLiD1(**A**) and rHNC (**B**). The binding site is indicated by ellipses. The electrostatic charges were calculated with APBS program in the UCSF Chimera [11].

Binding and Interaction Predictions of SM and LPC with rLiD1 and rHNC

To analyze the possible interactions of SM and LPC when bound to rLiD1 and rHNC, protein-ligand docking assays were performed using AutoDock 4.0 [12]. Molecular docking allows prediction of a potential binding mode and specific interactions between the proteins' residues and the ligand. A region that included the catalytic (residues 44 to 62), variable (residues 166 to 175), and flexible loops (residues 198 to 207), and the Mg^{2+} ion, was chosen as the binding site to the assays. The selected region for docking was large enough to accommodate the ligands' long carbon tail.

SM achieved close predicted binding affinity for both rLiD1($\Delta G = -11.56$ kcal/mol) and rHNC ($\Delta G = -11.78$ kcal/mol) (Figure S3). The SM polar group found a similar conformation in both proteins, buried into the binding site (residues His12, Glu32, Asp34, His47, Pro50, Cys51, Asp52, Cys53, Asp91, Lys93, Pro134, Tyr135, Asp164, Ser166, Tyr228, Trp230, Thr199, Cys201) (Figure S3A and C). In rLiD1, the polar group formed hydrogen bond interactions with His47 (one of the catalytic histidines) and Asp52, both in the catalytic loop, and with Lys93 (Figure S3B). Attractive charge interactions with the Mg^{2+}ion, Asp91 (involved in ion coordination) and Lys93 were also predicted. In rHNC, SM formed hydrogen bond interactions with Lys93 and Thr199 (Figure S3D). Interestingly, the trimethylamine group from SM was pointed to the inside of the pocket in rHNC, while it was more solvent exposed in the pose in rLiD1, allowing the formation of interactions with more residues in rHNC. Therefore, besides the contacts with the Mg^{2+}ion, Asp91, and Lys93, also present in rLiD1, interactions with Glu32 (involved in ion coordination), Asp164 and Trp230 were observed in the SM pose of rHNC. The interacting residues Glu32, His47, Asp34, Asp91, Lys93, and Trp230, previously known to affect substrate affinity and ion coordination [13], are conserved in both structures. The aliphatic tail did not find a common conformation between proteins, which might show the flexibility of this group, as expected due to their high solvent exposure.

Similar to SM, predicted binding affinity for LPC was close between rLiD1 ($\Delta G = -7.20$ kcal/mol) and rHNC ($\Delta G = -7.91$ kcal/mol) (Figure S4A,C). Although the polar group of LPC was inside the binding site, its conformation in the rLiD1 structure was less buried than in rHNC, with the trimethylamine group of the rLiD1-LPC complex once again solvent exposed. This small discrepancy ensured that the rHNC-LPC complex achieved attractive interactions with the Mg^{2+}ion, and residues Glu32, Asp91, Lys93, Asp164 and Trp230. While for rLiD1, the trimethylamine group interacted only with the Mg^{2+}ion, Asp91, and Lys93. Although the aliphatic tail of LPC is smaller, it was still solvent exposed, without specific interactions with the proteins. Therefore, similarly to the observed SM, this region was not anchored at any defined region of the receptors.

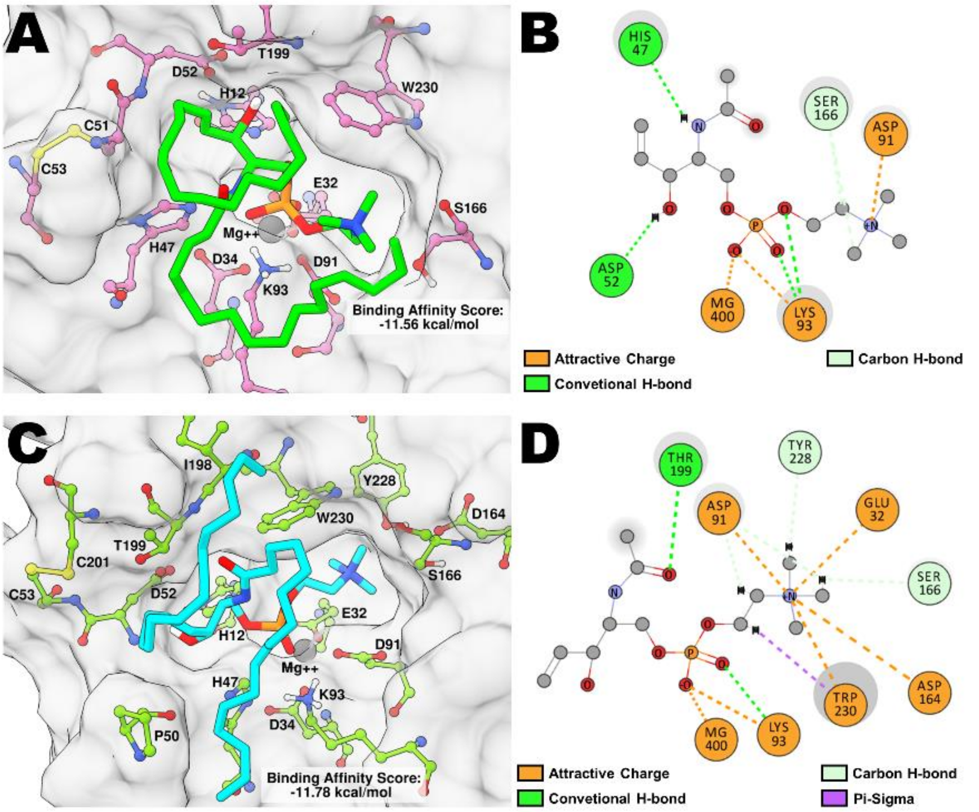

Figure S3. Predicted binding modes for SM in rLiD1 (**A** and **B**) and rHNC (**C** and **D**) from molecular docking. Interaction 2D maps were generated for the polar group of SM in both rLiD1 (**B**) and rHNC (**D**). (**A**) The polar group of SM in rLiD1 was buried into the binding pocket formed by the displacement of the flexible loop towards the catalytic loop and in close contact with the metal ion. (**B**) This group formed hydrogen bond interactions with His47, Asp52, and Lys93. Attractive charge interactions were also formed with the Mg^{2+} ion, Asp91, and Lys93. Carbon hydrogen bond was achieved with Ser166. (**C**) Similar to LiD1, the polar group of SM in rHNC was buried into the binding pocket and in close contact with the metal ion. (**D**) The polar group of SM displayed hydrogen bond interactions with Lys93 and Thr199. Docking of SM in the rHNC active site produced more attractive contacts than with rLiD1. Attractive charge interactions were achieved with the Mg^{2+} ion, Glu32, Asp91, Lys93, Asp164, and Trp230. Carbon hydrogen bond was found with Ser166 and Tyr228. The 3D surface figures were done with UCSF Chimera [11], while 2D maps were obtained with Discovery Studio Visualizer [14].

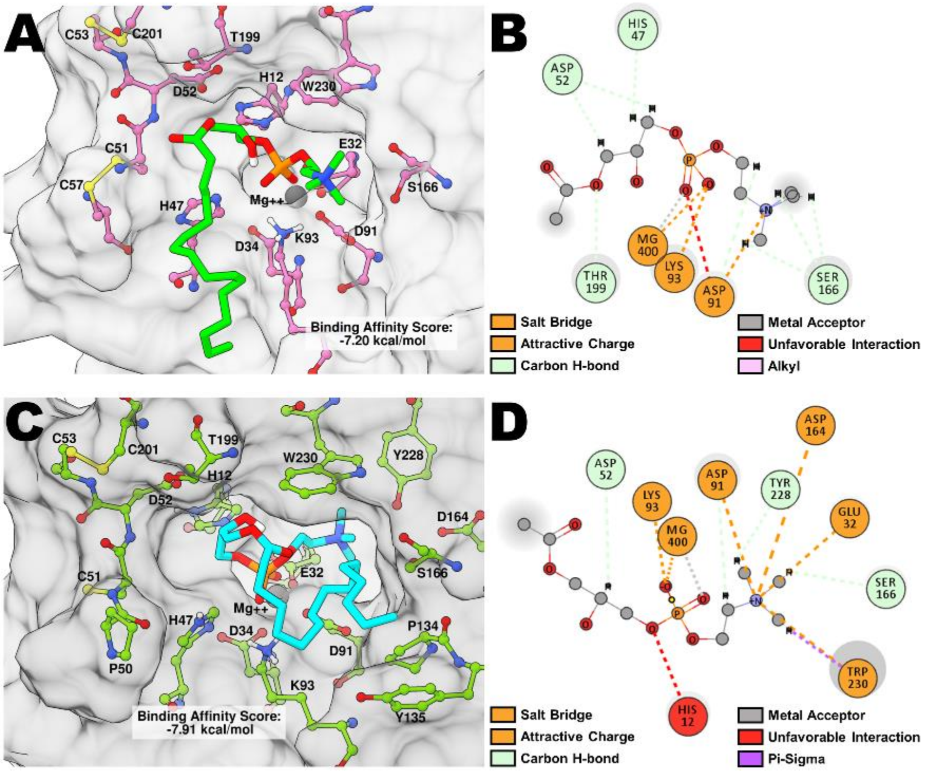

Figure S4. Predicted binding modes for LPC in rLiD1 (**A**) and rHNC (**C**) from molecular docking. Interaction 2D maps were generated for the polar group of LPC in both rLiD1 (**B**) and rHNC (**D**). (**A**) The polar group of LPC was buried into the binding pocket and in close contact with the metal ion. (**B**) This group displayed salt bridge interaction with Lys93 and carbon hydrogen bond interactions with His47, Asp52, Thr199, and Ser166. Attractive charge interactions were also formed with the Mg^{2+} ion, Asp91, and Lys93. (**C**) Similar to rLiD1, the polar group of LPC in rHNC was buried into the binding pocket formed and in close contact with the metal ion. (**D**) The LPC polar group achieved hydrogen bond interactions with Lys93. Docking of LPC in the rHNC active site produced more attractive contacts than the same substrate in rLiD1. Attractive charge interactions were formed with the Mg^{2+}ion, Glu32, Asp91, Lys93, Asp164 and Trp230. Carbon hydrogen bond was found with Asp52, Ser166, and Tyr228. The 3D surface figures were done with UCSF Chimera [11], while 2D maps were obtained with Discovery Studio Visualizer [14].

Dynamic Behavior of rLiD1 and rHNCStructures in Apo and Bound Simulations

Due to limitations in the docking methodology, which considers a single protein conformation, we expect that differences in the stability of the docking binding modes and interactions may arise as a consequence of the rHNC distinct sequence and residue changes near the binding site when compared to rLiD1. Therefore, we performed MD simulations to consider the flexibility of the proteins. First, we analyzed the RMSD progression against the initial modeled structures during 100 ns MD simulation to see if the proteins experienced major conformational changes. All simulations were stable after 40 ns of simulation time (Figure S5). Although flexible regions were observed, in solvent-exposed loops (Figure S6), no major conformation changes were identified from the simulations. However, when root-mean-square fluctuation (RMSF) was analyzed, small differences in residue fluctuations were observed. The increase of flexibility of the catalytic loop and the so-called flexible loop in the unbound rHNC, when compared to unbound rLiD1, might be associated with the number of residue substitutions in both regions (Figure S7A). There are seven and three substitutions in the catalytic and flexible loops, respectively, in rHNC when compared to rLiD1 (Table S3). On the other hand, the loop that comprises Asp91 and Lys93 was more rigid in rHNC. In this region, there are eight residue substitutions in rHNC (Supporting Info Table S3). This same loop trend could be observed in the bound, SM (Figure S7B) and LPC (Figure S7C) simulations.

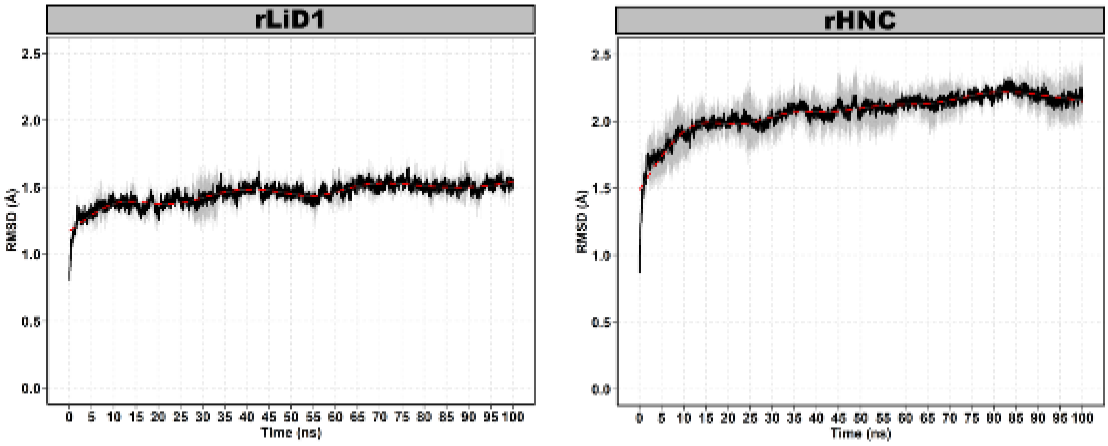

Figure S5. Average Cα-RMSD plots for all three simulations (apo, SM and LPC bound) of rLID1 and rHNC. The average of the three curves is displayed in black, standard deviation is in gray and the trend line of the curves is the red dashed line. Plots were done with the R program [15].

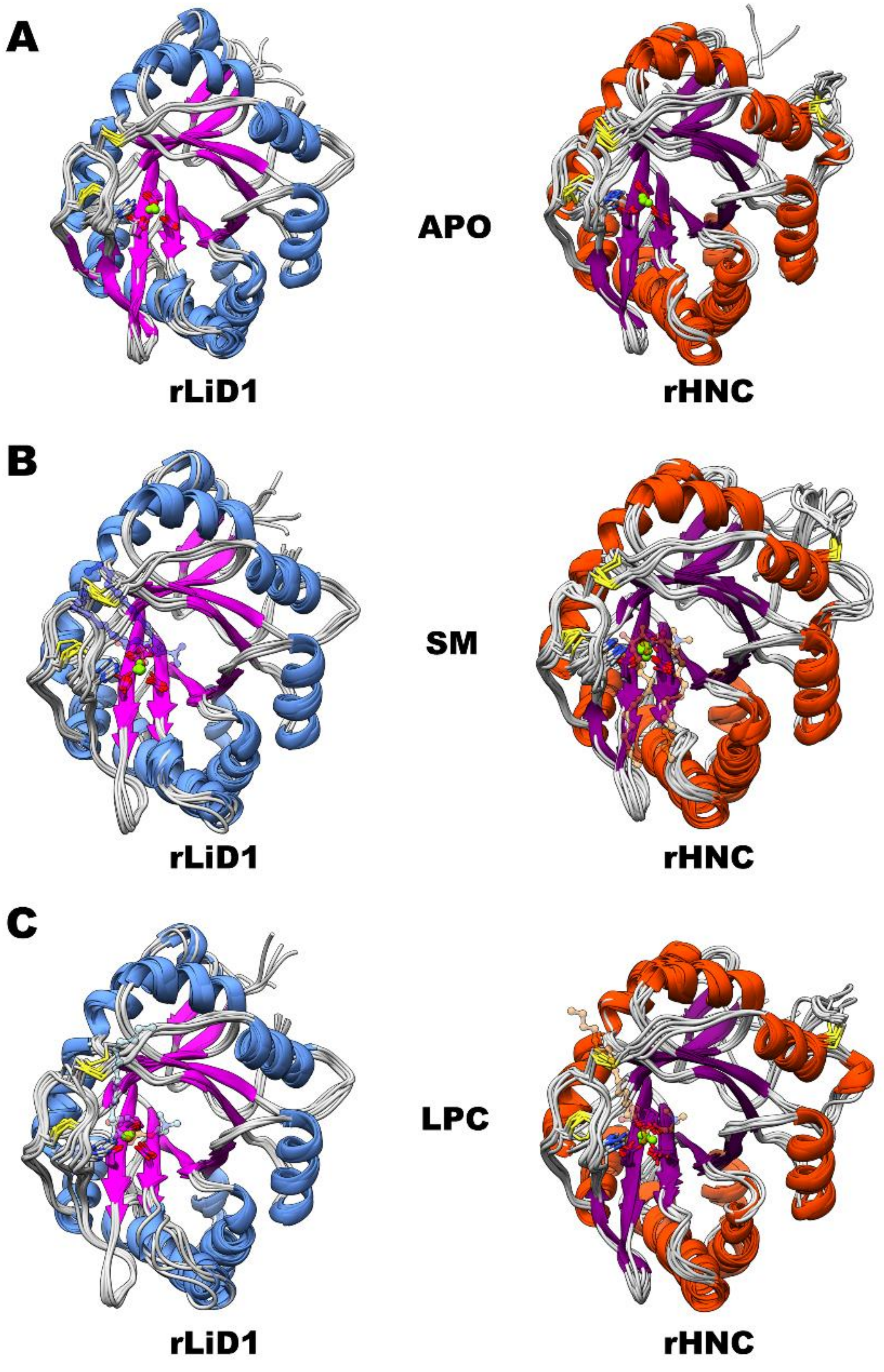

Figure S6. Superimposed representative structures obtained from clustering the simulation trajectories of each rLiD1 and rHNC system. (**A**) Structures from the unbound simulations. (**B**) Structures from the SM bound simulations. (**C**) Structures from the LPC bound simulations. Three dimensional representations were built with the UCSF Chimera [11].

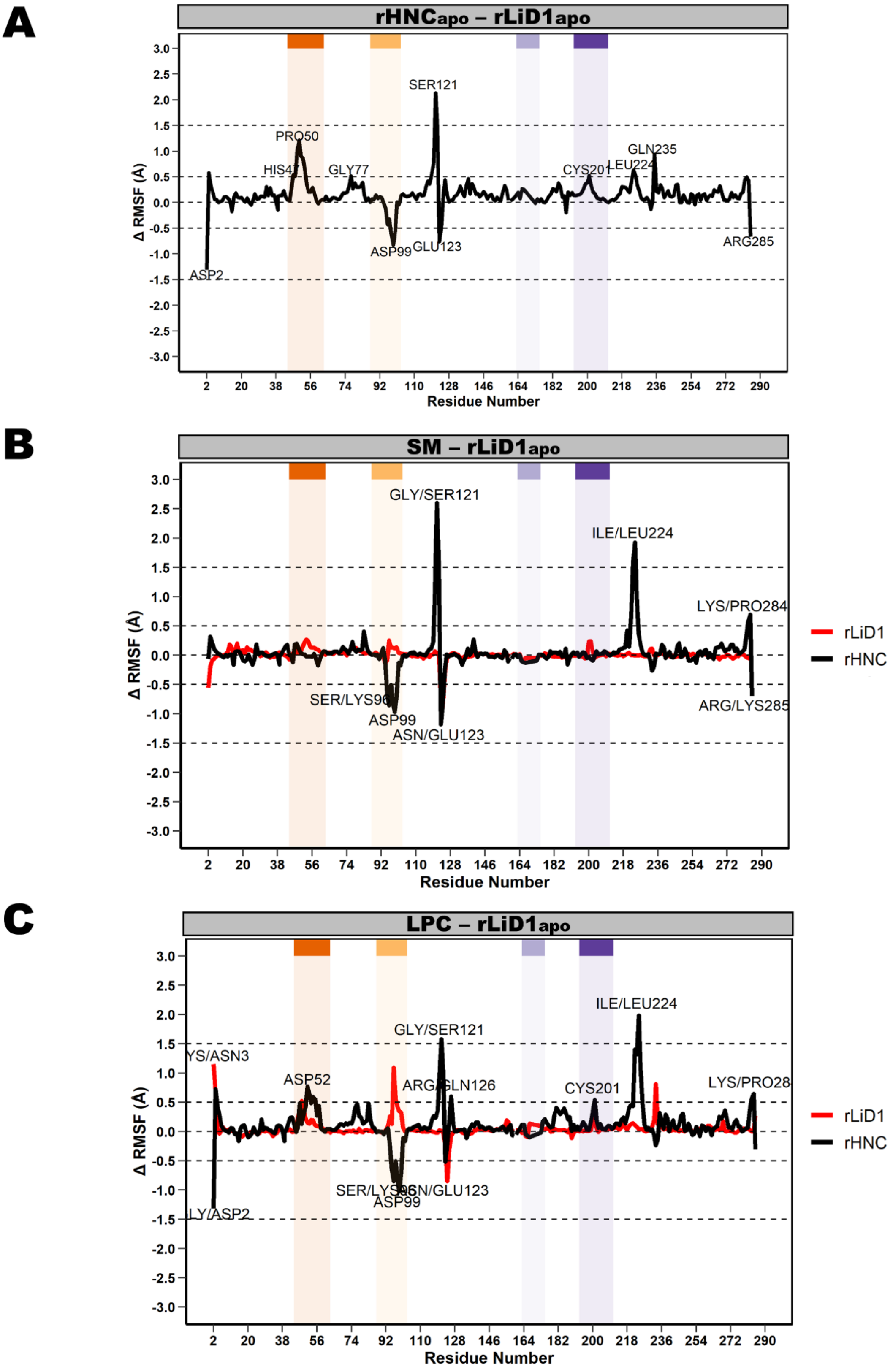

Figure S7. Per-residue fluctuation in apo and bound rLiD1 and rLiD1 simulations, using the unbound rLiD1 simulation as reference ($\Delta RMSF = RMSF_{simulation} - RMSF_{rLiD1apo}$). Positive values display an increase in flexibility of the residue when compared to the rLiD1 simulation. Negative values display a decrease in flexibility when compared to the reference simulation. The colored regions depict the catalytic loop (residues 44 to 62, orange), Asp91 loop (residues 87 to 103, light orange), variable loop (residues 166 to 175, light purple), and flexible loop (residues 198 to 207,

purple). **A**) The difference in fluctuation of the unbound structures (ΔRMSF = $RMSF_{rHNCapo}$ − $RMSF_{rLiD1apo}$) showed increase in flexibility of the catalytic, variable and flexible loops. While, the Asp91 loop became more rigid. **B**) The difference in fluctuation in the simulations of rLiD1 and rHNC bound to SM, using as reference the unbound rLiD1 simulation (ΔRMSF =$RMSF_{rLid1sm}$ − $RMSF_{rLiD1apo}$ and ΔRMSF =$RMSF_{rHNCsm}$ − $RMSF_{rLiD1apo}$). While the complex rLiD1-SM was close to the unbound simulation, the Asp1 loop of the rHNC-SM complex remained rigid. **C**) The difference in fluctuation in the simulations of rLiD1 and rHNC bound to LPC, using as reference the unbound rLiD1 simulation (ΔRMSF =$RMSF_{rLid1lpc}$ − $RMSF_{rLiD1apo}$ and ΔRMSF =$RMSF_{rHNClpc}$ − $RMSF_{rLiD1apo}$). While the complex rLiD1-SM was close to the unbound simulation, the Asp1 loop of the rHNC-SM complex remained rigid. LPC increased the flexibility of the catalytic and flexible loops in both proteins. However, once again the Asp91 loop had inverse flexibility, in rLiD1 simulation it was more flexible, whereas in the rHNC simulation it was more rigid. Plots were done with the R program [15].

Dynamic Behavior of Substrates Bound to rLiD1 and rHNC Structures

The substrates diverged significantly from the initial docking position in the bound simulations. SM was flexible in both rLiD1 (average RMSD of 9.8 ± 1.5 Å) and rHNC(average RMSD of 4.9 ± 1.09 Å) binding sites (Figure S8A). This flexibility was due to the mobility of the aliphatic tail, since the polar group equilibrated to a position and remained close to it throughout the simulation (Figure S8B). However, the SM polar group bound into rLiD1 deviated more from the docking position (average RMSD of 2.8 ± 0.3 Å) than when bound to rHNC (average RMSD of 1.2 ± 0.3 Å), due to the accommodation of the trimethylamine group from solvent-exposed in the docking pose to point to the inside of the binding pocket (Figure S8C). On the other hand, in the rHNC-SM simulation this group remained pointed to the binding site as in the docking mode (Figure S8D).

Although the aliphatic tail is smaller in LPC, the substrate also had a flexible behavior (Figure S9A) when bound to rLiD1 (average RMSD of 10.7 ± 1.9 Å) and rHNC(average RMSD of 8.4 ± 1.8 Å). LPC's polar group diverged from the initial docking mode more in rHNC (average RMSD of 2.6 ± 0.5 Å) than in rLiD1 (average RMSD of 1.7 ± 0.2 Å) (Figure S9B). Even with this flexible behavior, the trimethylamine group of the rHNC-LPC remained pointed to the inside of the binding site throughout the simulation as displayed in the initial docking pose (Figure S9D). In the rLiD1-LPC complex, the initial solvent-exposed trimethylamine group achieved a buried conformation that persisted throughout the simulation (Figure S9C). These results indicate that, despite an initial difference in solvent-exposure of the trimethylamine group in the docking results, for the four systems analyzed the most stable orientation of this group is pointing towards to binding site, where it is stabilized by multiple interactions.

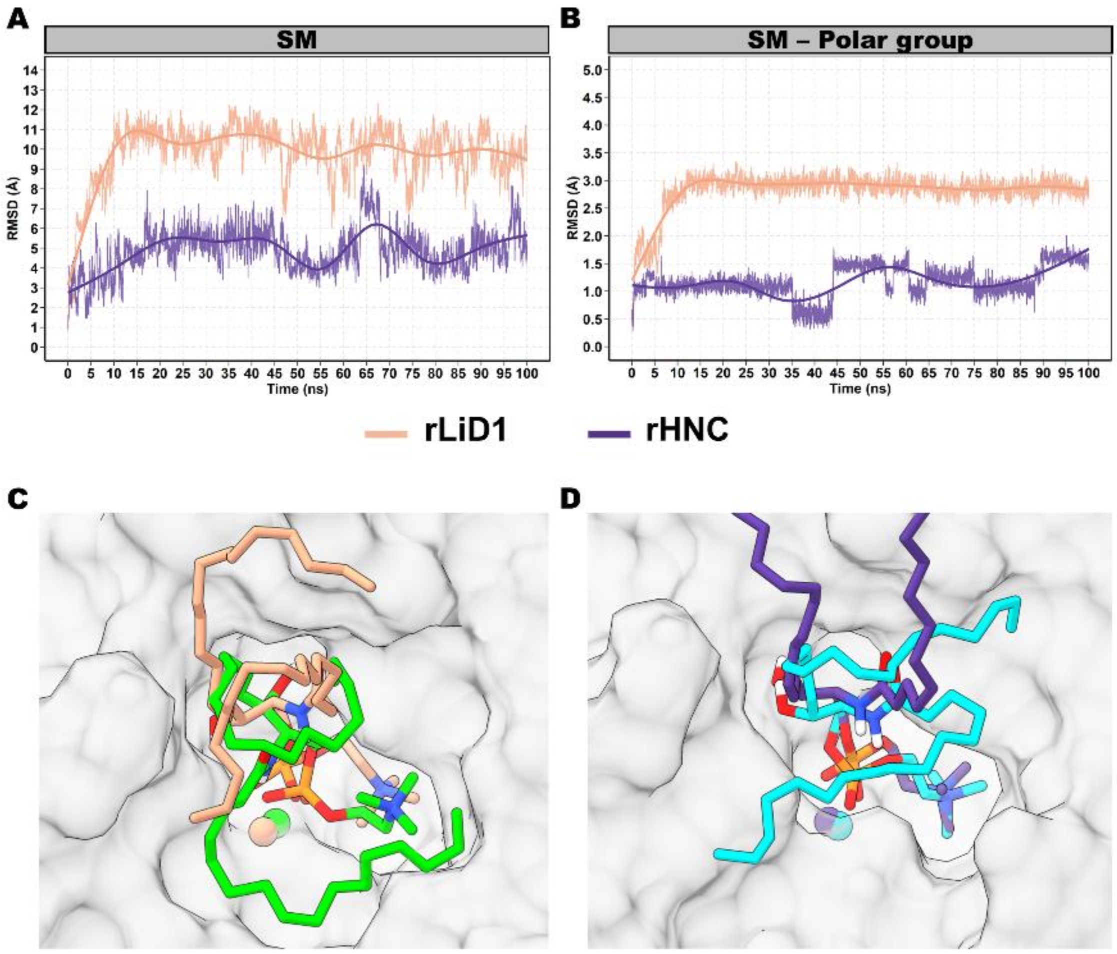

Figure S8. SM deviation throughout the rLiD1 and rHNC bound simulations. Representative structures of the most sampled ligand conformations, calculated by clustering the trajectories, are displayed compared to docking pose. (**A**) RMSD plot of SM when compared to the initial docked pose in both proteins. SM complexed to rLiD1 was more flexible than when bound to rHNC. (**B**) RMSD plot of the polar group of SM. The SM polar group bound to rLiD1 deviated more from the docking position than when bound to rHNC. (**C**) Accommodation of the trimethylamine group from solvent exposed (green) to buried inside of the binding pocket (salmon). (**D**) In the rHNC simulation, the trimethylamine group remained pointed to the binding site in the simulation (purple) similar to the docking binding mode (cyan). Plots were done with the R program [15] and three-dimensional representations were built with the UCSF Chimera [11].

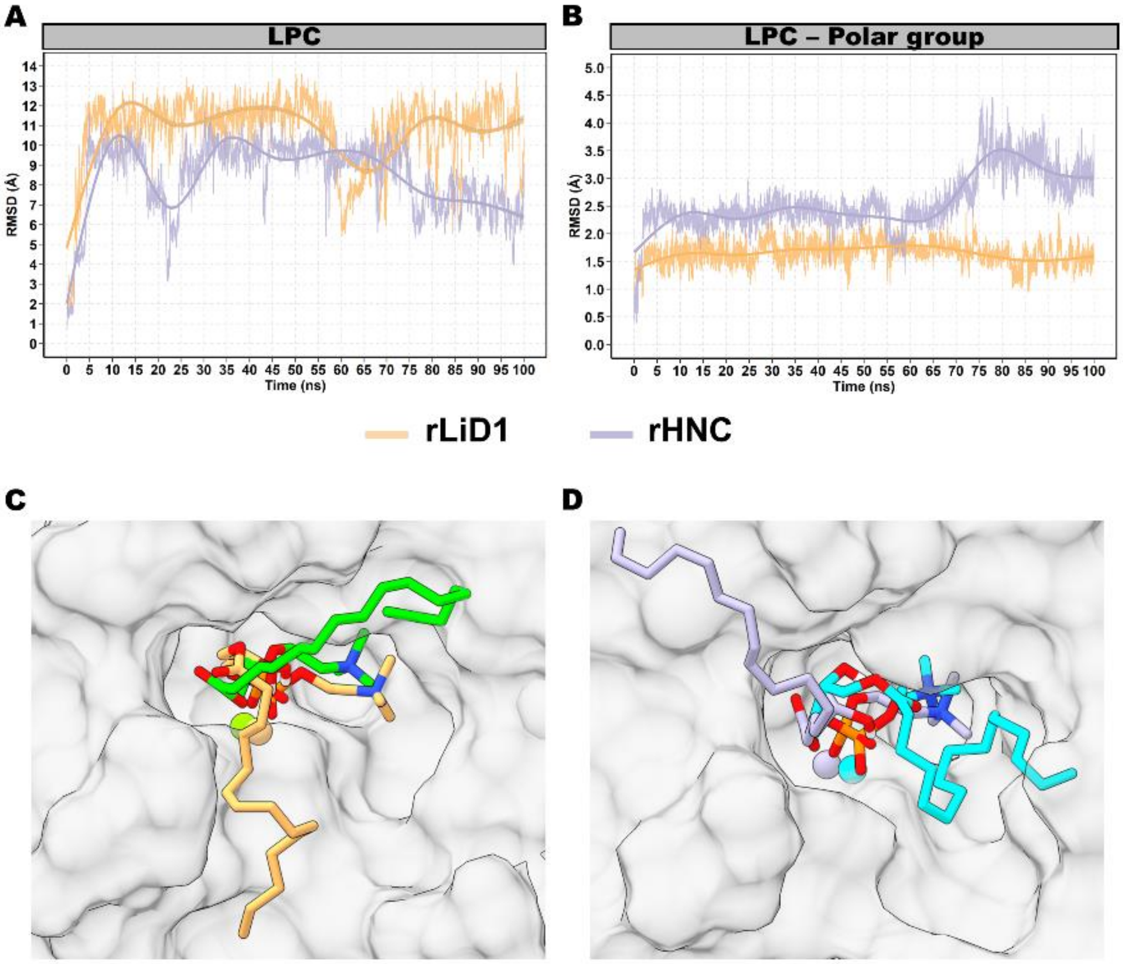

Figure S9. LPC deviation throughout the rLiD1 and rHNC bound simulations. Representative structures of the most sampled ligand conformations, calculated by clustering the trajectories, are displayed compared to docking pose. (**A**) RMSD plot of LPC when compared to the initial docked pose in both proteins. Overall, LPC complexed to rLiD1 was more flexible than when bound to rHNC. (**B**) The RMSD plot of the polar group of LPC showed that the substrate deviated more from its initial position in rHNC than when bound to rHNC. (**C**) Accommodation of the trimethylamine group from solvent exposed (green) to buried inside of the binding pocket (light orange). (**D**) In the rHNC simulation, the trimethylamine group remained pointed to the binding site in the simulation (purple) similar to the docking binding mode (cyan). Plots were done with the R program [15]and three-dimensional representations were built with the UCSF Chimera [11].

```
rLiD1        1 ---AGNRRPIWIMGHMVNAIGQIDEFVNLGANSIETDVSFDDNANPEYTY     47
                  ...:||||.:||||||:.||:||::||||::|.||:||||.||::||
rHNC         1 DSQEDKKRPIWNIGHMVNAVKQIEEFLDLGANALEADVTFDDNGNPKWTY     50

rLiD1       48 HGIPCDCGRNCKKYENFNDFLKGLRSATTPGNSKYQEKLVLVVFDLKTGS     97
               ||.||||.|:|.::|..:::||.:|..|:||:||:::..:|::.|||...
rHNC        51 HGTPCDCFRDCLRWEYVDEYLKRIRELTSPGSSKFRKGFILLMLDLKISK    100

rLiD1       98 LYDNQANDAGKKLAKNLLQHYWNNGNNGGRAYIVLSIPDLNHYPLIKGFK    147
               |.||..:.|||::|..:::..|:......:.|||||.|.:|....::.|:
rHNC       101 LSDNAKSKAGKEIADMIIKRLWSGSGEKAQLYIVLSFPYVNDIEFVRAFR    150

rLiD1      148 DQLTKDGH-PELMDKVGHDFSGNDDISDVGKAYKKAGITGHIWQSDGITN    196
               :::...|. .|...::|.|.|||:|:..:..||:|.|||.::||||||||
rHNC       151 ERVKSKGFASEAEKRIGWDISGNEDLGKIRDAYQKLGITDNVWQSDGITN    200

rLiD1      197 CLPRGLSRVNAAVANRDSANGF--INKVYYWTVDKRSTTRDALDAGVDGI    244
               ||.|...|:..||..|||...:  :.|||||||||:|:.::||..||||:
rHNC       201 CLTRSHDRLAEAVCKRDSDKEWPSLKKVYYWTVDKQSSMKEALKVGVDGM    250

rLiD1      245 MTNYPDVITDVLNEAAYKKKFRVATYDDNPWVTFKK-----    280
               :||.||.:..||||  :....|:|..:|:||....:
rHNC       251 ITNDPDDLVAVLNE--FSGTHRLANINDSPWQKIPRPKSNC    289
```

Figure S10. PairwiseSequence alignment between rLiD1 (UniProtKB/Swiss-Prot: P0CE81.1) and rHNC (UniProtKB/Swiss-Prot: A0A1L4BJ98) using Needleman-Wunsch algorithm. rLiD1 epitopes [16] are highlighted in yellow (similar to rHNC) and blue (different from rHNC). Conserved amino acids between the two sequences are also highlighted: same amino acid (|), amino acids with similar properties (:) and amino acids with weakly similar properties (.).

References

1. De Giuseppe, P.O.; Ullah, A.; Silva, D.T.; Gremski, L.H.; Wille, A.C.; Chaves Moreira, D.; Ribeiro, A.S.; Chaim, O.M.; Murakami, M.T.; Veiga, S.S.; et al. Structure of a novel class II phospholipase D: Catalytic cleft is modified by a disulfide bridge. *Biochem. Biophysic. Res. Commun.***2011**, *409*, 622–627.
2. Murakami, M.T.; Fernandes-Pedrosa, M.D.F.; De Andrade, S.A.; Gabdoulkhakov, A.; Betzel, C.; Tambourgi, D.V.; Arni, R.K. Structural insights into the catalytic mechanism of sphingomyelinases D and evolutionary relationship to glycerophosphodiesterphosphodiesterases. *Biochem. Biophys. Res. Commun.* **2006**, *342*, 323–329, doi:10.1016/j.bbrc.2006.01.123.
3. Murakami, M.T.; Fernandes-Pedrosa, M.F.; Tambourgi, D.V.; Arni, R.K. Structural basis for metal ion coordination and the catalytic mechanism of sphingomyelinases D. *J. Biol. Chem.* **2005**, *280*, 13658–13664.
4. Webb, B.; Sali, A. Comparative protein structure modeling using MODELLER. *Curr. Protoc. Bioinform.* **2014**, *47*, 5–6.
5. Colovos, C.; Yeates, T.O. Verification of protein structures: Patterns of nonbonded atomic interactions. *Protein Sci.* **1993**, *2*, 1511–1519.
6. Structural Analysis and Verification Server, SAVES; https://servicesn.mbi.ucla.edu/SAVES (accessed on 5 September 2019).
7. Eisenberg, D.; Lüthy, R.; Bowie, J.U. VERIFY3D: Assessment of protein models with three-dimensional profiles. *Methods Enzymol.***1997**, *277*, 396–404, doi:10.1016/S0076-6879(97)77022-8.
8. Benkert, P.; Künzli, M.; Schwede, T. QMEAN server for protein model quality estimation. *Nucleic Acids Res.***2009**, doi:10.1093/nar/gkp322.
9. SWISS-MODEL, Structure Assessment; https://swissmodel.expasy.org/assess(accessed on 5 September 2019)
10. ProSA-web: interactive web service for the recognition of errors in three-dimensional structures of proteins; https://www.came.sbg.ac.at/prosa.php (accessed on 5 September 2019)
11. Pettersen, E.F.; Goddard, T.D.; Huang, C.C.; Couch, G.S.; Greenblatt, D.M.; Meng, E.C.; Ferrin, T.E. UCSF Chimera—A visualization system for exploratory research and analysis. *J. Comput. Chem.* **2004**, *25*, 1605–1612.

12. Morris, G.M.; Huey, R.; Lindstrom, W.; Sanner, M.F.; Belew, R.K.; Goodsell, D.S.; Olson, A.J. AutoDock4 and AutoDockTools4: Automated docking with selective receptor flexibility. *J. Comput. Chem.* **2009**, *30*, 2785–2791.
13. Vuitika, L.; Chaves-Moreira, D.; Caruso, I.; Lima, M.A.; Matsubara, F.H.; Murakami, M.T.; Takahashi, H.K.; Toledo, M.S.; Coronado, M.A.; Nader, H.B.; et al. Active Site Mapping of *Loxosceles* Phospholipases D: Biochemical and Biological Features. *Biochim. Biophys. Acta* **2016**, *1861*, 970–979.
14. Biovia, D.S. Discovery Studio Visualizer; 2019. https://discover.3ds.com/discovery-studio-visualizer(accessed on 14 August 2020).
15. Development Core Team R (2013) R: A Language and Environment for Statistical Computing, reference index version 2.15.1 R foundation for statistical Computing, Vienne. Available online: https://www.r-project.org/ (accessed on 7 November 2019).
16. Ramada, J.S.; Becker-Finco, A.; Minozzo, J.C.; Felicori, L.F.; Machado de Avila, R.A.; Molina, F.; Nguyen, C.; de Moura, J.; Chávez-Olórtegui, C.; Alvarenga, L.M. Synthetic peptides for in vitro evaluation of the neutralizing potency of *Loxosceles* antivenoms. *Toxicon* **2013**, *73*, 47–55.
